# Tumor-Targeted Delivery Therapy Based on PLGA Nanoparticles

**DOI:** 10.3390/jfb17050207

**Published:** 2026-04-22

**Authors:** Fang Wu, Yuan Gao, Yongjie Chi, Danyang Wang, Siqi Zhang, Ocean Cheung, Kai Zhao, Hongsheng Lu, Qi Chen, Yu Chen, Lianyan Wang, Yanhua Zhu

**Affiliations:** 1 School of Pharmacy, Heilongjiang University of Chinese Medicine, Harbin 150040, China; wufang198762@163.com; 2 Institute of Process Engineering, Chinese Academy of Sciences, Beijing 100190, China; gaoyuan@ipe.ac.cn (Y.G.); chiyongjie21@ipe.ac.cn (Y.C.); wangdanyang24@ipe.ac.cn (D.W.); zhangsiqi20010823@163.com (S.Z.); 3 University of Chinese Academy of Sciences, Beijing 100190, China; 4 Key Laboratory of Bio-Based Material Science &Technology, Northeast Forestry University, Ministry of Education, Harbin 150040, China; 5 The Ångström Laboratory, Nanotechnology and Functional Materials Division, Department of Engineering Sciences, Uppsala University, Box 534, SE-751 21 Uppsala, Sweden; 6 Zhejiang International Science and Technology Cooperation Base for Biomass Resources Development and Utilization, Taizhou Key Laboratory of Biomedicine and Advanced Dosage Forms, School of Life Sciences, Taizhou University, Taizhou 318000, China

**Keywords:** PLGA nanoparticles, tumor targeting, stimulus-responsive release, drug delivery

## Abstract

Poly(lactic acid-lactic acid) (PLGA) has demonstrated significant application potential in tumor-targeted drug delivery systems due to its excellent biocompatibility, degradability, and multifunctionality for loading various therapeutic agents. PLGA nanoparticles (NPs) can achieve targeted delivery to tumor cells through specific surface modifications and stimulus-responsive release mechanisms, significantly enhancing drug accumulation efficiency at tumor sites while reducing toxic side effects on normal tissues. This review systematically summarizes the fundamental physicochemical properties of PLGA materials and recent advances in tumor-targeting strategies for PLGA NPs. It comprehensively elucidates research breakthroughs in PLGA-based delivery systems regarding stimulus-response mechanisms, passive targeting, active targeting, and tumor combination immunotherapy, while revealing the intrinsic logic of synergistic strategies for enhancing targeting efficiency. Finally, from the perspective of clinical translation and individualized oncology, this review conducts an in-depth assessment of the current challenges and looks forward to future research directions, aiming to provide forward-looking guidance for the development of precision nanomedicine.

## 1. Introduction

Globally, there were about 10.4 million cancer-related deaths and 18.5 million new cases in 2023. If these statistics continue to increase at the current rate of 60.7% and 74.5%, respectively, by 2050, there will be 30.5 million new cases of cancer and 18.6 million deaths from the disease worldwide [1]. There are a number of contemporary therapies commonly used by physicians, including radiation, chemotherapy, and surgery, that have been shown to be effective in the treatment of cancer. However, complications after surgery and drug resistance, along with systemic toxicity, frequently limit the effectiveness of these traditional treatment modalities [2].

In recent years, the global field of cancer treatment has undergone profound paradigm shifts, with therapeutic approaches evolving from traditional single and homogeneous strategies toward precision medicine, combination therapies, and personalized treatment. Current malignant tumor management primarily encompasses multiple modalities including local treatment, systemic therapy, and adjuvant supportive care. Local treatment primarily involves surgical resection, radiotherapy, and minimally invasive interventional ablation, which can directly eliminate local lesions. Systemic therapy includes chemotherapy, targeted therapy, immunotherapy, endocrine therapy, and cell therapy, enabling systemic clearance of potential micrometastases. Contemporary clinical practice predominantly adopts combination treatment regimens such as surgery combined with postoperative chemoradiotherapy, concurrent chemoradiotherapy, or targeted/immunotherapy combined with chemotherapy. These strategies enhance antitumor efficacy, reduce toxic side effects, and improve patient outcomes through synergistic effects, establishing them as core clinical approaches in oncology treatment [3,4].

Despite the aforementioned progress, many therapeutic drugs still face challenges in achieving clinical translation due to issues such as poor solubility, low bioavailability, inadequate targeted accumulation, and systemic side effects. Nanomedicine provides robust solutions to address these challenges. Among various nanocarriers, polymer nanoparticles (NPs) can significantly improve pharmacokinetic properties, enhance tumor-targeted accumulation capacity, and achieve controlled drug release. Various synthetic polymers such as poly(D,L-lactic acid), poly(D,L-lactic acid-co-hydroxyacetic acid copolymer), poly(ε-caprolactone), polyamino acids, and natural polymers including alginate, chitosan, and gelatin have been applied in the field of drug delivery. The Poly(lactic acid-lactic acid) (PLGA) is a biodegradable polymer and has been classified as Generally Recognized as Safe (GRAS) by the U.S. Food and Drug Administration (FDA). PLGA has been widely used in the field of drug delivery due to its exceptional biocompatibility, biodegradability, and distinctive physicochemical properties [5,6,7,8,9,10].

PLGA NPs have gained attention in tumor drug delivery research due to their easy surface modification, varied drug carriers, and controlled degradation [11,12,13]. These NPs’ flexible design allows for precise modification in accordance with particular therapeutic requirements, improving drug efficacy and offering a solution for tailored targeted therapy. Drug degradation can be effectively prevented by encapsulating different chemotherapeutic agents in PLGA NPs. If the PLGA NPs are engineered to provide sustained release of the drug at the tumor site, they may improve the antitumor activity of the drug while also reducing the side effects associated with chemotherapy. Furthermore, the inclusion of specific targeting ligands that can preferentially bind to their corresponding receptors or biomarkers on the surface of cancer cells can enhance the delivery and efficacy of the drug when using PLGA NPs. This allows for precise drug concentration at tumor sites to improve therapeutic outcomes. Drug resistance can be successfully decreased, and the success rate of tumor treatment can be increased by combining the delivery of PLGA NPs with medications with various mechanisms of action. Also, when combination therapy is used, overall patient outcomes may be improved by overcoming the limitations associated with single-agent therapies [14,15,16].

PLGA NPs can be flexibly modified through multiple pathways, enabling passive targeting via enhanced permeability and retention effects, active targeting based on ligand-receptor interactions, and stimulus-responsive drug release within the tumor microenvironment. These modifications significantly improve drug accumulation efficiency at tumor sites while reducing toxicity to healthy tissues. The material is particularly suitable for delivering chemotherapeutic agents, targeted drugs, proteins, peptides, nucleic acids, and photosensitizers, making it an ideal carrier for combination therapies and integrated chemotherapy-immunotherapy strategies. This article systematically evaluates PLGA-based tumor-targeted delivery systems, focusing on their fundamental properties, active targeting strategies, passive targeting mechanisms, stimulus-responsive drug release behaviors, and their application progress in combined cancer immunotherapies. Finally, we analyze key challenges in clinical translation and provide forward-looking insights into the physicochemical design of PLGA NPs. for personalized treatment, aiming to advance the clinical translation and application of such delivery systems in precision cancer therapy.

## 2. Properties and Advantages of PLGA

### 2.1. Biocompatibility and Biodegradability of PLGA Material

**Figure 1:**
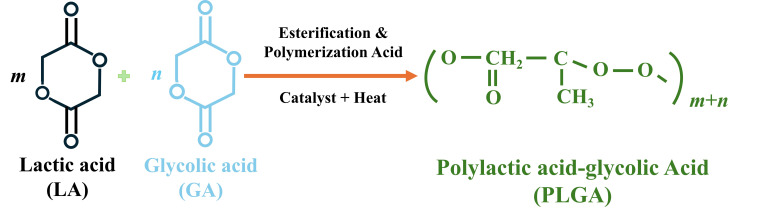
Schematic diagram of PLGA synthesis (*m* = number of lactate units, *n* = number of hydroxyacetic acid units).

PLGA is a biodegradable polymer formed by the esterification of lactic acid (LA) and glycolic acid (GA) monomers utilizing a specific ratio of both monomers. PLGA is a unique polymer with an ingenious combination of the properties of LA and GA. The polymerization process primarily involves the random ring-opening copolymerization of cyclic dimers (1,4-dioxane-2,5-dione) of LA and GA, forming ester bonds between the two acids during copolymerization, ultimately yielding a linear aliphatic polyester (Figure 1) [17,18,19,20]. Precise control of the LA:GA monomer ratio enables the preparation of various PLGA materials, thereby achieving precise regulation of PLGA degradation kinetics. In biomedical applications, PLGA copolymers with LA:GA ratios of 50:50, 65:35, 75:25, and 85:15 are most commonly employed [20], which can provide controllable degradation rates, excellent biocompatibility, and adaptability to different drug release requirements by adjusting the LA: GA ratio (Table 1).

PLGA will break down primarily via hydrolytic reactions, whereby water will enter the polymer matrix over a period of time, resulting in the cleavage of the ester bonds that make up PLGA and subsequently leading to the production of two small molecules, which are LA and GA. Both LA and GA are metabolites that are produced in the human body and can be eliminated by the tricarboxylic acid (TCA) cycle as carbon dioxide (CO_2_) or by urinary excretion of the acidic byproducts [25,26]. Because of these characteristics, PLGA materials generally will not generate excessive amounts of inflammation or an immune response when used in vivo environment, so the need for surgical removal of the implant will not exist, which will ultimately help to reduce patient suffering and risks. PLGA materials also support the adhesion, proliferation, and differentiation of cells. The surface characteristics of PLGA materials may be improved via physical and chemical modifications to most commonly improve compatibility with the specific cell types that are being used for tissue engineering and regenerative medicine approaches [6,27,28].

### 2.2. High Drug Loading Efficiency of PLGA Nanoparticles

PLGA has the potential to serve as a delivery vehicle for tumor immunotherapies through drug encapsulation, allowing for sustained release of the drug to target and stimulate an effective immune response against tumor cells while maintaining sufficient therapeutic levels over time. The ability to reduce the frequency of administration of medications through sustained release will enhance patient compliance and potentially improve treatment outcomes. Furthermore, encapsulation of drugs within PLGA NPs provides protection from degradation by enzymes, changes in pH or other physiological conditions in vivo, thus preventing premature degradation of these agents, thereby improving the stability and bioavailability of PLGA-based formulations [11,12,13,29].

Due to its unique physicochemical properties, PLGA exhibits excellent anchoring and encapsulation performance. By adopting different preparation processes, this material can effectively encapsulate drugs with diverse properties (Table 2). The choice of synthesis technology is highly dependent on the cargo’s hydrophilicity. For instance, single emulsion-solvent evaporation is the gold standard for encapsulating hydrophobic chemotherapeutics (e.g., paclitaxel, doxorubicin), providing stable loading within the hydrophobic microdomains and preventing aqueous aggregation. Conversely, double emulsion or microfluidic nanoprecipitation techniques are employed for hydrophilic payloads, such as proteins, peptides, and nucleic acids.

PLGA NPs can also be designed based on the physicochemical properties of pharmaceuticals and therapeutic requirements. Using ester bond chemical coupling, researchers successfully conjugated doxorubicin with terminal groups of PLGA to prepare doxorubicin-PLGA NPs. In vitro release experiments demonstrated that these conjugated NPs achieved sustained slow release of doxorubicin over one month, while NPs loaded with free doxorubicin exhibited rapid release characteristics within five days. This strategy significantly extended the release duration of doxorubicin [42]. Through chemical modification, such as the introduction of amino groups to generate a positively charged membrane layer, the surface charge density of PLGA NPs surface can be easily regulated, thereby enhancing electrostatic interactions between negatively charged surface nucleic acid drugs (e.g., siRNA, mRNA) [43], thereby significantly increasing the encapsulation efficiency of nucleic acids. The amphiphilic properties of PLGA material provide hydrophobic microdomains for solubility of hydrophobic chemotherapeutic agents (i.e., paclitaxel, doxorubicin), stable loading, and prevention of drug aggregation in aqueous environments [44]. Additionally, the porosity and thickness of the membrane material can be precisely adjusted through preparation processes such as emulsification-solvent evaporation, which not only ensures adequate drug encapsulation but also establishes a gradient release barrier to achieve sustained or pulsed drug release at the tumor site to enhance therapeutic efficacy [45]. In addition, PLGA have great biocompatibility and can be made into a composite membrane structure with other materials such as liposomes and polyethylene glycol (PEG) to improve both their potential and capacity to deliver drugs [11,46].

Strategies to enhance drug loading efficiency into PLGA NPs include surface modification, optimization of preparation parameters, and supplementary methodologies. Neha et al. developed a combinatorial approach to treat breast cancer through loading paclitaxel (PTX) and gefitinib (GEF) into PLGA NPs to overcome the limitations of using each drug on its own. The initial study using a Chou-Talalay method was performed to find the best combination of the two drugs together in order to optimize the synergetic effect. With the use of a DoE approach, they were able to prepare, optimize, and properly characterize the NPs with an average diameter of 169.79 nm with encapsulation efficiencies of PTX and GEF encapsulated within them equal to 89.17% and 76.89%, respectively (*w*/*w*) [47]. Zheng et al. successfully manufactured lyophilizable diPTX-SS NPs using flash nano-precipitation with very long stability (over one month) as well as a high loading capacity (91% by weight) [48].

### 2.3. Stimulus-Responsive Release of PLGA Nanoparticles

#### 2.3.1. Mechanism and Kinetics of Physiological Drug Release

In physiological environments, the drug release behavior of PLGA NPs primarily exhibits a three-phase release profile [49,50]. The first phase is an initial burst release triggered by drug surface adsorption, resulting from the physical adsorption of drug molecules on the NPs surface followed by rapid desorption upon contact with body fluids. This phase typically occurs during the early release stage and may lead to a sharp increase in drug concentration. The second phase involves diffusion-controlled release mediated by the polymer matrix, where drug molecules diffuse through the pores of the PLGA matrix or inter-polymer chain spaces. The release rate is influenced by diffusion coefficients, concentration gradients, and matrix structure, and this process can persist for extended durations. The third phase is degradation-controlled release induced by ester bond hydrolysis. Under physiological conditions, PLGA polymers undergo hydrolysis, leading to ester bond cleavage and gradual matrix degradation, thereby releasing the encapsulated drug. The drug release rate is closely correlated with polymer degradation kinetics, with the relative contributions of these mechanisms depending on polymer composition, molecular weight, particle size, and the physicochemical properties of the drug itself [51,52].

**Figure 2:**
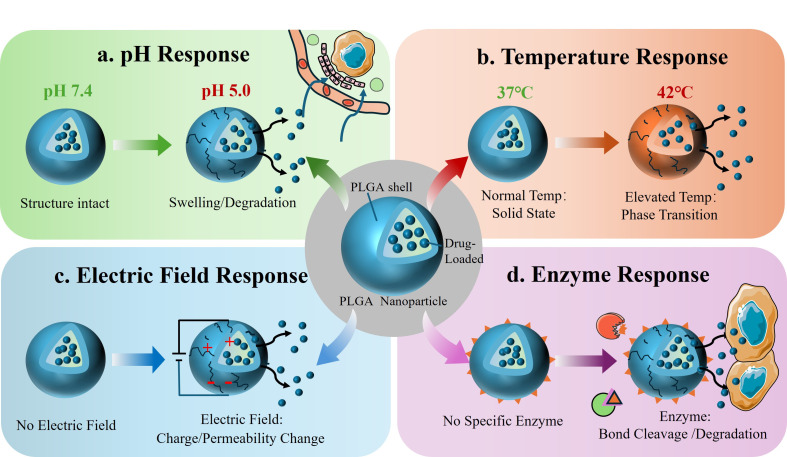
(**a**) pH response: Low pH promotes the hydrolysis of PLGA, thereby accelerating drug release. (**b**) Temperature response: Phase transitions induced by localized high temperatures or targeted heating enhance drug release. (**c**) Electric field response: External electric fields modulate NPs permeability or charge changes to drive drug release. (**d**) Enzyme response: Tumor-associated enzymes-mediated site-specific bond cleavage facilitates drug release at pathological sites. The arrows in the figure indicate the process of drug release from the NPs in response to different stimuli.

Furthermore, through appropriate modification of PLGA, it can respond to external stimuli (such as pH, temperature, light, etc.), thereby achieving controlled drug release in vivo [53,54,55,56]. This approach not only enhances therapeutic efficacy but also significantly reduces its toxic side effects (Figure 2). A thorough understanding of these dominant physiological release mechanisms is of paramount importance before further designing stimulus-responsive drug delivery systems.

#### 2.3.2. pH Responsiveness

The degradation rate of PLGA is significantly correlated with the pH value of the surrounding environment. The hydrolysis rate of PLGA increases in low-pH environments (such as the tumor microenvironment), which speeds up polymer chain cleavage and facilitates drug release. Al-Hosani et al. created a straightforward technique to create biocompatible and biodegradable pH-responsive hybrid NPs in order to address problems with the poor circulation stability and ineffective targeting of conventional NPs. In order to reduce nonspecific interactions with serum proteins and macrophages that impede target recognition, these NPs are composed of Doxorubicin-Triphenylphosphonium (Dox-TPP)-loaded PLGA core covalently wrapped in a cross-linked bovine serum albumin (BSA) shell. The BSA shell is further modified with an acidity triggered rational membrane (ATRAM) peptide, enabling its specific internalization into cancer cells within the acidic tumor microenvironment [57]. The cumulative release percentage of Dox-TPP in each experimental group was monitored over a 24 h time scale, with specific data presented in Table 3 below.

#### 2.3.3. Temperature Responsiveness

When PLGA is blended with other thermosensitive polymers such as poly(N-isopropylacrylamide), its temperature-responsive properties can be introduced. At particular temperatures, this combination can change the material’s phase behavior, allowing for precise drug release. The structure of poly(N-isopropylacrylamide), a common temperature-sensitive substance, includes both hydrophilic amide groups (-CONH_2_) and hydrophobic isopropyl groups [-CH(CH_3_)_2_]. Water molecules occupy the many voids in the polymer’s three-dimensional network structure at low temperatures. These water molecules create a layer of water molecules on the surface of the polymer by forming hydrogen bonds with the amide groups. Water is expelled and gel is formed when the temperature rises because the hydrogen bonds are broken, the isopropyl groups dehydrate, the water content drops, and the hydrophobic groups strengthen their association. When the temperature exceeds the critical gelation temperature, the solubility of the polymer changes, resulting in controlled drug release from the PLGA carrier that encapsulates the drug [58,59]. For instance, in a study, researchers employed the nanoscale precipitation method to encapsulate the photothermal agent tetra(4-carboxyphenyl)porphyrin (TCPP) with the anticancer drug isothiocyanate isothiocyanate (Iso) within PEG-b-PLGA polymer NPs for breast cancer treatment. In vitro release experiments conducted at pH 7.4 and 37 °C demonstrated that TCPP and Iso exhibited similar release profiles, releasing 58% and 42%, respectively, within the initial 12 h. Subsequently, photothermal conversion experiments were performed using 650 nm laser irradiation to observe temperature changes in deionized water. Results showed that the temperature rise curve increased with NPs concentration: the solution temperature elevated from 26 °C to 42 °C at a 30 μg/mL TCPP concentration, whereas the deionized water group exhibited only a 1.5 °C increase. Additionally, the TCPP-Iso conjugated NPs demonstrated cancer cell-killing efficacy at relatively mild temperature elevations under laser irradiation, exhibiting high synergistic effects, while the NPs showed no significant toxicity to normal tissue cell lines [60].

#### 2.3.4. Magnetic Responsiveness

The PLGA carrier can be made to react to changes in magnetic or electric fields. The internal structure of NPs and the drug release process can be affected by the application of external electric or magnetic fields [61,62]. In order to deliver proteins to bone marrow-derived primary dendritic cells (BMDCs), Ritprajak et al. looked into a delivery system that combined magnetic fields with a biocompatible PLGA copolymer and superparamagnetic iron oxide NPs (SPION-PLGA NPs). In vitro cell experiments using the RAW 264.7 cell line were conducted to evaluate the cellular uptake capacity of TAMRA-labeled SPION-PLGA NPs under an applied magnetic field of 260 mT. Imaging results obtained by transmission electron microscopy and confocal laser scanning microscopy demonstrated that the cellular uptake efficiency of NPs was significantly higher in the presence of magnetic fields compared to the control group without magnetic fields. Under an external magnetic field, the generated SPION-PLGA nanocomposites demonstrated superparamagnetism, minimal cytotoxicity, and effective uptake by macrophages and BMDCs. The nanocomposites with BMDCs were used in an immunomodulatory experiment. By increasing the expression of MHC II, CD80, and CD86, this SPION-PLGA carrier in conjunction with an external magnetic field can greatly improve BMDC maturation. This strategy’s ability to induce an immune response was confirmed by a notable increase in the production of IFN-γ and IL-12 [63].

#### 2.3.5. Enzyme Responsiveness

Through the inclusion of specific enzyme characteristics in PLGA, the release of drugs in response to enzymes can be achieved. To assist in this process, utilizing proper enzymes and conducting logical chemical design can improve the sensitivity and specificity of the PLGA material; therefore, enhancing the efficacy of a drug delivery system [64]. The tumor microenvironment frequently overexpresses the enzyme protease B. When it binds to PLGA, its particular substrate peptides can achieve precise enzyme-triggered release. The linker is broken down by protease B in tumor cells, which causes nanoparticle disintegration and quick drug release into the cytoplasm. This greatly increases the drug’s cytotoxic effect on tumor cells. By conjugating gadolinium chelate (Gd-DOTA) to a peptide-hyaluronic acid hybrid via a cathepsin B-responsive linker (GFLG), Guo et al. created a therapeutic nanomedicine (Dendronized-HA GFLG-Gd/PTX-PLGA NPs). In vitro release experiments were conducted by incubating nanoparticles (NPs) at 37 °C and pH 5.4, with the addition of papain exhibiting bioactivity similar to cysteine protease B. Concurrently, the release efficiency of PTX was evaluated under conditions without papain, pH 7.4, or pH 5.4. Results demonstrated that PTX could be effectively released from nanoparticles in the presence of papain at pH 5.4, with approximately 54% release within 1.5 h and about 80% release after 24 h. However, in the absence of papain at pH 5.4, PTX release was negligible (1.47%). Under pH 7.4 conditions, only minimal PTX release (15%) was observed after 48 h incubation. In vivo experiments indicated that overexpressed cathepsin B cleaves GFLG in the tumor microenvironment, releasing Gd-DOTA for enhanced magnetic resonance imaging (MRI) and paclitaxel (PTX) for chemotherapy. The nanomedicine significantly improved MRI contrast and showed superior antitumor efficacy in a 4T1 breast tumor model [65].

## 3. Tumor-Targeting Strategies

### 3.1. Passive Targeting

**Figure 3:**
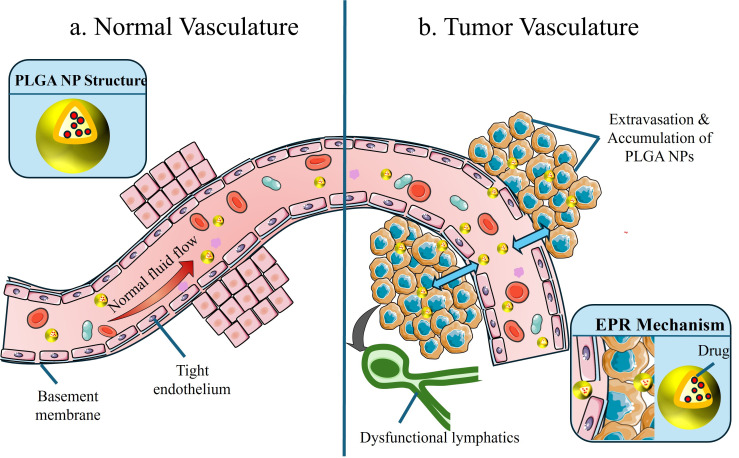
The EPR effect: A physiological basis for passive tumor targeting. The illustration contrasts (**a**) Normal Vasculature, characterized by an intact basement membrane and tight endothelium that prevents the leakage of nanoparticles (NPs), with (**b**) Tumor vasculature, which exhibits leaky junctions and dysfunctional lymphatic drainage, leading to a disorganized microenvironment that enables extravasation and selective accumulation of PLGA-based NPs in tumor tissues. The red arrows in the figure represent the normal fluid flow, the blue arrows indicate that PLGA NPs accumulate towards tumor cells, and the black arrows show that the tumor causes dysfunction of the lymphatic system.

The intrinsic size of NPs and the distinct anatomical and pathophysiological anomalies of tumor vasculature, such as the EPR effect, are exploited by passive targeting [66,67]. Rapid tumor tissue growth causes vascular endothelial cells’ intercellular connections to loosen, creating gaps that range in diameter from 100 to 1000 nm. Concurrently, PLGA NPs with sizes between 50 and 200 nm can penetrate the vascular wall and remain in the tumor stroma for prolonged periods of time due to the tumor region’s underdeveloped lymphatic drainage system [68,69]. The schematic diagram of the EPR effect is shown in Figure 3.

Researchers have also improved the efficiency of passive targeting by optimizing the hemodynamic properties of PLGA NPs through the surface modification of PEG. PEG chains can create a hydration layer on the surface of the particle, preventing non-specific binding with plasma proteins and decreasing phagocytic clearance from the mononuclear phagocyte system (MPS) which leads to an increase in circulation time of the NPs [70]. For example, researchers used a gelatin (GelMA) hydrogel system prepared with ibrutinib-loaded PLGA-PEG-folic acid NPs (IBT-PPF-NPs) and octreotide PLGA microspheres (OCT-PLGA-MPs) to explore the use of localized drug delivery systems in treating glioblastoma multiforme (GBM). The localized nature of this drug delivery system creates a high-drug concentration in the tumor area, thereby improving the therapeutic effect. The in vitro cell studies indicated that IBT-PPF-NPs inhibited glioma cell proliferation in a concentration-dependent manner, while the octreotide was not cytotoxic; however, together they created an additive antitumor effect. The in vivo studies suggested that the drug-loaded hydrogel significantly inhibited tumor growth. The MRI and histological studies showed that the IBT-PPF-NPs treatment increased caspase-3 level and decreased Ki-67 labeling of glioma cells, while the octreotide inhibited tumor proliferation by inhibiting angiogenesis and decreasing CD31 [71]. The surface charge of PLGA NPs also influences passive targeting efficacy: a neutral or slightly negatively charged surface reduces electrostatic repulsion with negatively charged vascular endothelial cells, facilitating better penetration into the tumor stroma [72]. Also, the efficiency of the Enhanced Permeability Retention (EPR) phenomenon is different between tumors due to differences in the way that tumors generate blood vessels (angiogenesis); there is considerable variability (spatially and qualitatively) in density of the endothelial cells, the structural integrity of the basement membrane, and the presence of lymphatics between separate tumors in different patients [68,73]. Other factors, such as patient age, degree of inflammation present, and how many prior treatments a patient has received also affect vascular permeability. As a result, there is considerable variability in the EPR effect among different patients.

Although the EPR effect provides a theoretical foundation for passive targeting strategies, clinical translational studies have revealed significant limitations. Comprehensive meta-analyses indicate that the typical median tumor delivery efficiency of systemic NPs (including PLGA NPs) is generally below 1% (typically approximately 0.7% of the injected dose), with specific values influenced by tumor type and nanoparticle design parameters. This phenomenon is also observed in carriers such as liposomes and inorganic NPs, highlighting universal challenges in the field of nanomedicine. The gap between preclinical research findings and clinical practice is primarily attributed to multiple biological barriers, including rapid clearance by the mononuclear phagocyte system, dense tumor extracellular matrix, and elevated interstitial fluid pressure, which severely impede NPs penetration into deep tumor tissues. To overcome these critical challenges in NPs design, a shift from reliance on passive accumulation strategies to the development of advanced bioresponsive PLGA systems is essential. Such systems should enable active navigation and effective penetration of highly heterogeneous tumor microenvironments, thereby enhancing drug accumulation efficiency at tumor sites [74,75,76,77,78].

### 3.2. Active Targeting

**Figure 4:**
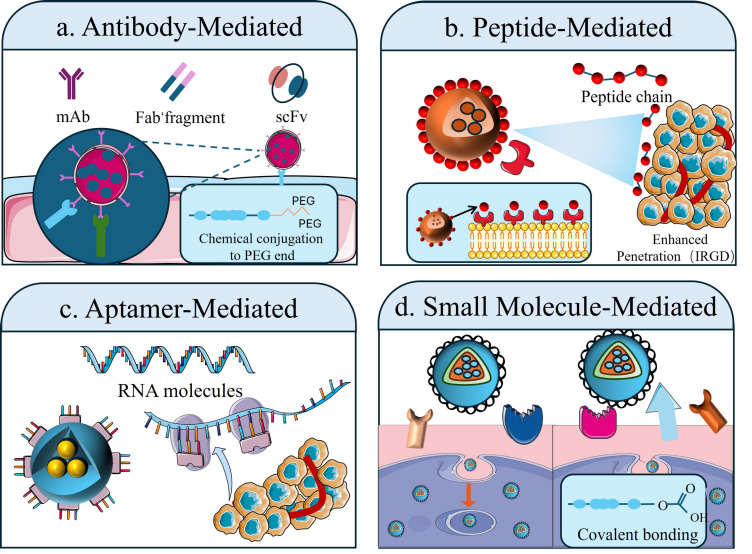
Classification of Ligand-Mediated Active Targeting Strategies. Active targeting enhances tumor-specific accumulation by binding various ligands to the surface of nanoparticles (NPs). Methods (**a**–**d**) correspond to antibody-mediated, peptide-mediated, aptamer-mediated, and small molecule-mediated targeting strategies, respectively, which hold significant applications in active tumor-targeting therapy based on PLGA NPs.

To develop targeted delivery systems that effectively deliver chemotherapeutic agents (or other therapeutic agents) with high precision into tumor cells, it is crucial to have targeted ligands that trigger specific binding to and uptake by tumor cells or their tumor microenvironments. To accomplish this goal, targeted ligands are conjugated to the surfaces of PLGA NPs to promote specific binding, thus increasing the accumulation of drugs in tumor cells significantly while decreasing nonspecific uptake in normal tissues. The selection process for active targeting ligands should be based on antigens or receptors expressed highly on tumor cell surfaces. Common ligands that are currently utilized in both research and in the clinic are antibodies, peptides, aptamers, polysaccharides, and small biomolecules (Figure 4) [79,80,81,82,83,84,85,86]. Ligand-mediated active tumor-targeted therapies exhibit the potential for maximizing therapeutic efficacy, and, therefore, minimizing systemic side effects; thus, they are emerging as a novel, and potentially critical, platform for safe and effective treatment of cancer.

#### 3.2.1. Mediated by Antibodies/Antibody Fragments

Antibodies (Abs) have unique characteristics of both high specificity and high affinity for their respective antigens with dissociation constants ranging from nanomolar. They are composed of two long heavy-chain sections and two shorter light-chain sections, typically. In certain cases, targeting ligands can be designed in the variable regions of antibodies, significantly reducing the overall molecular weight and mitigating adverse immune responses [83,87,88,89,90,91,92]. If whole Ab or Ab fragments are then conjugated to the surfaces of PLGA NPs, precise targeted delivery will occur as a result of the high affinities and specificities of Abs for recognizing the tumor cell antigens on the surface of tumor cells [93,94,95,96].

Monoclonal antibodies (mAbs), are widely regarded as one of the most well-known ligands that are used in active targeting because of the high specificity and affinity of mAbs. Conjugating mAbs to PLGA NPs produces NPs that can actively seek out tumor cells by recognizing antigens on the surface of the tumor cells using the complete antibody molecule attached covalently to the surface of the PLGA nanoparticle [90,91]. Castro et al. developed a novel active targeted delivery system for treating lung cancer by investigating lipopolysaccharide hybridized PLGA NPs loaded with docetaxel (DCX) and by functionally conjugating the anti-Tn antigen monoclonal antibody (Chi-Tn mAb) to the NPs. In vitro studies demonstrated that Chi-Tn mAb modification significantly increased the endocytosis of the NPs in A549 cells and substantially decreased the viability of A549 cells. The in vivo research showed that the DCX-loaded LPHNPs that were targeted by Chi-Tn mAb significantly inhibited the growth of the tumor and improved the survival of tumor-bearing mice relative to the free drug [96]. However, because of the large molecular weight of the functional antibodies (about 150 kDa), this may create steric hindrance on the surface of the NPs and therefore affect the hemodynamics of the NPs. Thus, due to their unique properties, antibody fragments with lower molecular weights (15–50 kDa) that have greater penetrability into tissues, such as Fab (fragment antigen binding) and scFv monovalent antibodies (single-chain variable fragment), have gradually gained interest [88,95].

The scFv fragment is composed of the heavy chain variable region (VH) and light chain variable region (VL) linked by a flexible peptide chain, which preserves the intact antigen-binding activity of the antibody while reducing non-specific interactions [89]. Giglio et al. created a nanoparticle-based engineered system to target c-MET expressing cells, the NPs were created using a conjugation of an in-house developed (3H3-His C scFv) engineered scFv and PLGA NPs that allowed for precise and directed delivery of the anti-cancer agent verteporfin (VP) into A549 lung carcinoma cells. The engineered scFv maintained its binding ability and structural stability. Successful functionalizing of the NPs and a drug loading capacity of 3 µg of VP per mg of NPs. Investigations demonstrated a statistically significantly increased specific uptake of the NPs in the A549 cells [94].

#### 3.2.2. Peptide Ligand-Mediated

Peptide ligands have become an important class of ligands in active targeted delivery systems due to their small molecular weight, ease of synthesis, and low immunogenicity [97,98]. During chemotherapy, peptides can disrupt key tumor signalling pathways, induce apoptosis in tumor cells and provide effective inhibition of tumor angiogenesis, clearly demonstrating the utility of peptides as multi-functional molecules that both target and regulate all aspects of tumor development [98,99,100]. In immunotherapy, peptides can act as antigen-presenting molecules that activate appropriate immune response or function as immune checkpoint inhibitors that allow for the reactivation of T-cells which have been inactivated by the tumor [101,102]. By loading peptides onto PLGA NPs, they can be targeted to specific overexpressed receptors located on the surfaces of tumor cells, facilitate the active uptake of NPs and significantly improve the accuracy of drug delivery to the tumor [103,104,105]. Integrin-targeting peptides have been identified as one of the most studied types of peptide ligands used for the surface modification of PLGA NPs [106].

Tumor vascular endothelial cells and solid tumors (such as breast, lung cancer, and melanoma) have a high expression of integrin (αvβ3). Furthermore, cyclic RGD peptides have the ability to target and connect to integrin, making them ideal targeting ligands [106,107,108,109,110,111]. Yadav and colleagues developed RGD receptor-targeted PLGA NPs for the controlled and targeted co-delivery of cisplatin (CDDP) and upconversion NPs (UCNP) in lung cancer therapy. PLGA NPs were made using the double emulsion process, and Pluronic F127-RGD conjugate was prepared through the chemical method of carbodiimide. The pharmacokinetic data and assessment of safety in BALF were performed in a rat animal model. The data showed that the RGD-targeted PLGA NPs had a controlled-release therapy for 72 h. Additionally, pharmacokinetic data showed that RGD-targeted NPs had an efficacy of 4.6× that of cisplatin (CDDP)-50 [109].

Antimicrobial peptides (AMPs) can also be used as targeted ligands in tumor therapy due to their ability to interact with anionic phospholipids at the surface of the tumor cells [112,113,114,115]. A recent study loaded Polybia-derived MP-1 peptides into PLGA NPs. The study has demonstrated that MP-1 can directly interact with programmed cell death ligand 1 (PD-L1), preventing PD-L1 from interacting with programmed cell death receptor 1 (PD-1) which restores T cell activity and blocks the immunosuppressive effect of T cells. This mechanism inhibits the ability of cancer to evade the immune system and enhances attacks from immune cells on cancer cells, representing a novel therapeutic approach to treat triple-negative breast cancer (TNBC) [116].

#### 3.2.3. Adapter-Mediated

Adaptors are single-stranded DNA or RNA molecules created through the selection of an exponential ligand enrichment system evolution (SELEX) technology. Adaptor molecules exhibit high-affinity and specific binding to targets (including tumor-specific receptors, proteins, or small molecules) and adaptors possess properties including low molecular weight, low immunogenicity, and ease of modification [117,118,119,120,121], making them an ideal candidate for developing active targeted delivery systems using PLGA. Attaching aptamers chemically to the surface of PLGA NPs provides a means for receptor-mediated endocytosis utilizing the aptamers’ ability to bind specifically to tumor cell surface receptors, which greatly enhances drug delivery and concentration within tumor cells [122,123]. For instance, recent researchers have developed a self-regulating DNA nanostructure CRISPR-Cas12a system-Tatna (Triple-Lock Cascade Tumor Therapy Nanocapsule)-for efficient targeted tumor therapy. The Tatna system comprised the following components: (1) a high-drug-concentrated functionalized DNA tetrahedral structure (DT); (2) a Cas12a/crRNA ribonucleoprotein complex (Cas12a RNP); and (3) the chemotherapeutic agent doxorubicin (DOX). Using a multi-site activation cascade response, Tatna produced highly directional drug delivery and potentiated the antitumor effect of drugs. Aptamer incorporation directed against nuclear localizing regions allows Tatna to target and efficiently uptake tumor cells. The DNA components encapsulated within pH-sensitive PLGA nanocapsules maintain stability for DOX and Cas12a in the bloodstream and facilitate a controlled release within an acidic tumor microenvironment. Additionally, overexpressed mRNA APE1 within tumor cells activates the Cas12a RNP, promoting disassembly of the DT structure, facilitating the release of both DOX and Cas12a into the nucleus, thus leading to the death of cancer cells [124]. This self-regulating multifunctional strategy vastly improves the efficacy of chemotherapy, while also minimizing the potential for off-target activity.

RNA-based therapy is a major potential area for treatment for cancer patients, providing the basis for several new treatment approaches, including tumor-type vaccination, protein replacement therapies, cell therapies and gene therapies [125,126]. One way of achieving targeted and efficient tumor-specific accumulation of siRNA is through the use of targeted NPs. In one study, for instance, siRNA targeting VAV1 (siVAV1) was encapsulated in a PLGA NP conjugated to an ApoB-derived peptide ligand that showed high binding affinity for proteoglycans and low-density lipoprotein receptors overexpressed on pancreatic cancer (PC) cell surfaces and in the extracellular matrix (ECM). The inhibitory effects of siVAV1-delivered NPs (T-NPs) on tumor growth were mediated by a decrease in the levels of both VAV1 mRNA and protein expression. When T-NPs were delivered to mice with PC, the T-NPs showed superior tumor accumulation compared to untreated mice, leading to progressive inhibition of tumor growth and metastasis and improved survival compared to untreated PC mice [127].

#### 3.2.4. Biological Small Molecule Mediation

Small biomolecules (typically with molecular weights < 1000 Da) have emerged as a promising ligand class for active targeting systems in PLGA NPs due to their structural simplicity, low synthesis cost, excellent stability, and minimal immunogenicity. Small biomolecules have low steric hindrance, which allows them to bind efficiently to specific receptors on the tumor cell surface that are often overexpressed while not significantly impacting the amount of time that the NPs will stay in the bloodstream to be accurately delivered to their intended site. Folic acid (FA), galactose, and bisphosphonates are representative examples of small biomolecule ligands; targeting strategies to deliver compounds that bind to the FA receptor (FR) have been the subject of extensive research [128,129,130,131,132,133]. The FR is present in greater amounts on the surfaces of a variety of solid tumors (e.g., 100–300 times greater expression in the cells of ovarian cancer, breast cancer, and lung cancer) compared to normal cells, and accordingly, there is little to no expression of FR in normal tissue, making FR an ideal target for folate-modified PLGA particles for these types of tumors [134,135,136]. For example, Zhang et al. have developed a novel folate (FA)-modified chitosan (CS)-polylactic acid-co-glycolic acid (PLGA)-nanoparticle carrier (CPSF) for the delivery of sorafenib to lung cancer cells. The CPSF displayed sustained (approx. 4% release rate over 2 h) and pH-dependent (at pH 5.0 approx. 18% cumulative release rate within 2 h) release properties. In vitro treatment of A549 cells with CPSF for 24 h resulted in a survival rate of 13%, compared to 78% for mesenchymal stem cells (MSCs). The expression levels of caspase-9 and p53 genes were upregulated by more than 8-fold and 11-fold, respectively, whereas the expression level of the Bcl-2 gene was reduced by 5-fold, confirming its selective cytotoxicity against cancer cells [134].

## 4. Application of PLGA NPs in Tumor Therapy

### 4.1. Encapsulation of Different Types of Drugs

#### 4.1.1. Chemotherapeutic Agents

PLGA has significantly expanded the clinical application prospects of various drugs due to its outstanding drug delivery capability. Currently, classical chemotherapeutic agents, including paclitaxel, doxorubicin, 5-fluorouracil, gemcitabine, and cisplatin, have been successfully encapsulated into PLGA NPs. Relevant studies have evaluated the antitumor efficacy of these nanocarriers through systematic in vitro and in vivo experiments [40,109,137,138]. For instance, researchers prepared gemcitabine as PLGA NPs In vitro drug release experiments combined with pharmacokinetic analysis demonstrated that nanoparticleized gemcitabine not only prolonged drug delivery duration and significantly improved bioavailability, but also achieved longer systemic circulation time compared to free gemcitabine, providing a novel nanodelivery platform for pancreatic cancer treatment [137]. In another study, researchers prepared a 5-fluorouracil-PLGA NP (Anti-EGFR-5-FU-PLGA-PEG-NP) targeting epidermal growth factor receptor and evaluated its in vitro antitumor activity using the human colon cancer cell line HCT 116. The results demonstrated that compared to 5-FU-PLGA-PEG-NP, Anti-EGFR-5-FU-PLGA-PEG-NP exhibited higher cellular uptake levels and stronger cytotoxicity, providing a novel approach for EGFR-positive colorectal cancer cells [139].

Enhancing the antitumor activity of existing drugs and further exploring the therapeutic potential of traditional medicines remain cutting-edge directions in scientific research. Encapsulating these drugs into PLGA NPs provides an innovative strategy for optimizing the delivery efficiency and clinical efficacy of various therapeutic compounds.

#### 4.1.2. Encapsulation of Cytokines

PLGA can also encapsulate small molecules in vivo, including inflammatory factors [140,141]. Interleukin-10 (IL-10) is a key anti-inflammatory mediator that protects the host from excessive pathogen response and plays critical roles in wound healing, autoimmune disorders, cancer, and homeostasis. For instance, a study employed a dual emulsion method to encapsulate recombinant IL-10 within the biodegradable polymer PLGA, preparing IL-10-PLGA NPs. Researchers conducted in vitro experiments using the J774A.1 macrophage cell line and in vivo experiments in BALB/c mouse models to evaluate the bioactivity of these NPs. The results demonstrated that compared to free IL-10, IL-10-PLGA NPs significantly prolonged the biological half-life, achieved sustained drug release, and reduced the production of pro-inflammatory cytokines interleukin-6 (IL-6) and tumor necrosis factor (TNF) [142]. Granulocyte-macrophage colony-stimulating factor (GM-CSF) has demonstrated clinical activity in cancer immunotherapy but faces challenges such as high systemic toxicity and low bioavailability. Researchers prepared GM-CSF-loaded PLGA/PLGA-PEG NPs using phase separation technology. Monocytic receptor activation experiments in mice confirmed that the released GM-CSF maintained its biological activity and structural integrity. Compared with the control group induced by IFN γ and LPS to generate M1 type macrophages, the nanoparticle-treated group did not induce significant transcriptional changes in inflammatory regulatory genes in BMDMs. This study lays a preliminary foundation for in vivo research on GM-CSF-loaded PLGA/PLGA-PEG NPs in tumor immunomodulation [143].

#### 4.1.3. Gene Therapy

Furthermore, PLGA NPs can regulate gene expression by delivering exogenous nucleic acids, thereby inhibiting tumor growth or inducing tumor cell death [144,145]. For instance, researchers designed an inhalable non-viral siRNA vector for lung cancer treatment by coating PLGA onto cationic lipid vesicles loaded with siRNA using microfluidic control technology, resulting in the preparation of shell-core-based polymer lipid hybrid nanoparticles (HNPs). The designed HNPs exhibit mucosal inertness, demonstrating significantly enhanced stability and favorable safety profiles in mucus and bronchoalveolar lavage fluid (BALF), while effectively improving mucus permeability and cellular uptake efficiency [146]. In addition, DNA methyltransferase (DNMT) inhibitors have achieved significant breakthroughs in the field of tumor therapy. Researchers for the first time encapsulated the DNMT1 inhibitor decitabine into spherical PLGA nanoparticles and coated their surfaces with PD-L1 antibodies and macrophage membrane (aMM) vesicles, ultimately synthesizing the Dec@PLGA@aMM complex. The Dec@PLGA@aMM complex significantly upregulated the expression levels of p14 and p16 genes in a concentration-dependent manner and enhanced tumor-suppressive effects by reversing the silencing of tumor suppressor genes (TSGs) caused by high DNA methylation. This novel targeted PLGA NP is designed to treat hepatocellular carcinoma by reducing DNA methylation levels and blocking the PD-L1 signaling pathway [147].

#### 4.1.4. Contrast Agent Encapsulation

As a biodegradable polymer, PLGA was demonstrated to have significant application potential in the field of integrated tumor diagnosis and therapy. By co-loading contrast agents and therapeutic drugs into the PLGA matrix, precise tumor imaging and targeted therapy can be achieved [148,149,150,151]. For instance, researchers have developed a tumor-targeted molecular probe for pancreatic cancer imaging. Through amideation reactions, the CKAAKN peptide was conjugated with PEG-PLGA, and ultra-small superparamagnetic iron oxide (USPIO) polymer magnetic NPs suitable for magnetic resonance imaging (MRI) were prepared using an emulsifying solvent evaporation method, labeled as USPIO@CKAAKN-PEG-PLGA. Cell uptake experiments revealed that the modification with CKAAKN peptide significantly enhanced the binding capacity of USPIO to CKAAKN-positive BxPC-3 cells compared to the untargeted control group. In vitro MR imaging studies showed that the targeted nanoparticles co-incubated with BxPC-3 and HPDE6-C7 cells exhibited significantly reduced signal intensity, providing a new direction for pancreatic cancer treatment [148]. In another study, researchers prepared PLGA nanoparticles (NPs) targeting paclitaxel loaded with actin-binding protein GSN (GSN-PTX-PLGA NPs). Experimental results demonstrated that GSN-PLGA NPs labeled with fluorescent dye DiI exhibited enhanced fluorescence aggregation in Hca-F cells and tumor-bearing mouse models. Additionally, GSN-PLGA NPs demonstrated favorable imaging performance in vitro, with echo intensity progressively increasing as the concentration of GSN-PLGA NPs elevated. These findings provide clinical experimental evidence and visual tracking methods for inhibiting tumor growth and lymph node metastasis [151].

### 4.2. Tumor Immunotherapy

#### 4.2.1. Tumor Vaccine Vector

PLGA vaccine therapy for tumors has emerged as a significant direction in tumor immunotherapy in recent years, with the core concept being the delivery of tumor antigens via PLGA nanocarriers to activate the immune system for tumor recognition and elimination [152,153,154,155]. For instance, in a recent study, researchers encapsulated tumor-targeting peptide TMTP1, dendritic cell (DC) receptor mannose, and adjuvant monophosphatidylcholine A (MPLA) into PLGA to prepare NPs (NP-TP1@M-M). The study demonstrated that NP-TP1@M-M could capture and enrich more tumor-specific antigens post-chemotherapy, stimulate DC maturation, activate adaptive immune responses, and when combined with immune checkpoint blockade therapy, maximize the release of the body’s immune potential, thereby providing an effective therapeutic strategy for ovarian cancer (OC) treatment [152]. In a study, researchers utilized MHC Ihigh, CD80high, and CD86high dendritic cell-like cells combined with PLGA to prepare a personalized autologous nanovaccine for immunotherapy of postoperative metastatic cancer. This nanovaccine significantly enhanced antigen delivery efficiency to lymphoid organs and improved antigen presentation efficiency through tumor cell self-presenting mechanisms, thereby breaking through the conventional vaccine development paradigm [154].

#### 4.2.2. Delivery of Immune Checkpoint Inhibitors

The therapeutic efficacy of immunotherapy is limited, partially attributed to rapid drug clearance rates and associated nonspecific toxicity [102,156,157,158]. The application of immune checkpoint inhibitors delivered via PLGA nanoparticles in tumor therapy represents an innovative strategy that integrates immunotherapy with nanomedicine delivery systems, aiming to enhance therapeutic outcomes, reduce toxic side effects, and achieve tumor-specific targeting. In one study, researchers prepared α-PD-L1 F(ab)-PEG-PLGA nanoparticles (α-PD-L1 NP) by linking the truncated Fc segment of α-PD-L1 monoclonal antibody (α-PD-L1 mAb) to a PEG-PLGA polymer. Compared to α-PD-L1 mAb, α-PD-L1 NP exhibited significant advantages in promoting tumor cell uptake and reducing self-aggregation due to its surface charge. Additionally, α-PD-L1 NP reduced renal excretion and phagocytosis by mononuclear phagocyte systems, thereby prolonging its retention time in the host system while demonstrating more pronounced inhibitory effects on MC38 tumor growth [158]. In another study, researchers developed a nanodelivery platform using PLGA to encapsulate PD-L1 small interfering RNA (siRNA) and PD-1 siRNA. Results from mouse models carrying TC-1 and EG7 tumors demonstrated that PLGA (PD-L1 siRNA + PD-1 siRNA)-NP enhanced host immune responses by restoring CD8^+^ T cell function and promoting cytotoxic CD8^+^ T cell responses, with significant tumor growth inhibition compared to the free control group [157].

#### 4.2.3. Modulation of Tumor Microenvironment

The TME not only provides an optimal “niche” for tumor cell proliferation but also exhibits unique physicochemical barriers that significantly hinder the penetration of conventional drugs. Researchers utilized thioketal (TK) as a linker to conjugate PEG with PLGA, constructing a PEG-TK-PLGA carrier. Subsequently, Atovaquone (Ato) and Cabozantinib (Cabo) were encapsulated into this carrier to prepare Ato/Cabo@PEG-TK-PLGA NPs. These NPs were delivered transdermally to melanoma sites via a gel spray system containing the transdermal enhancer borneol. The results demonstrated that high levels of reactive oxygen species (ROS) in the tumor microenvironment rapidly cleave TK bonds, thereby triggering the release of Ato and Cabo from nanoparticles, effectively reversing the hypoxic and immunosuppressive tumor microenvironment [159]. In addition, activation of the interferon gene stimulator (STING) signaling pathway in the tumor microenvironment has been demonstrated to induce robust anti-tumor immune responses. However, achieving therapeutic efficacy typically requires frequent intra-tumoral injections over several months, posing significant challenges for clinical translation. To address this, researchers encapsulated STING agonists within PLGA to prepare NPs. Experimental studies in mouse tumor models revealed that a single intra-tumoral injection of STING agonist nanoparticles elicited potent local and systemic anti-tumor immune responses compared to free STING agonists, effectively inhibiting tumor growth and significantly prolonging survival [160].

### 4.3. Adjuvant Combination Therapy

In many cancer treatments, researchers have shown that combining different types of therapies can greatly improve the effectiveness of treating tumors. For example, combination therapies target the unique vulnerabilities of tumor cells and can simultaneously affect more than one pathway, such as inhibiting angiogenesis, inducing apoptosis, or enhancing immune responses, which allows for the destruction of more tumor cells and a greater chance that the tumor will not become resistant to the drug. These combination therapies improve the quality of life for the cancer patients and increase the chances of surviving long-term, which leads to a more tailored and efficient method of treatment. Studies illustrate that combination therapies increase the amount of tumor shrinkage from the first round of treatments and lower the rate risk of the tumor reoccurring; therefore, providing more complete treatment options for the patients [161,162,163,164,165].

Photodynamic therapy (PDT) can kill the tumor cell and potentially also stimulate an immune response that will attack the tumor. However, because PDT alone does not usually provide a very good immune response, some researchers have investigated the use of an immune checkpoint inhibitor with PDT to enhance the therapeutic response to PDT [166,167,168,169,170,171,172]. For example, researchers loaded the photosensitizer ZnF16Pc with indoleamine 2,3-dioxygenase (IDO) inhibitor NLG919 onto ferritin and PEG-PLGA NPs, respectively. In this composite delivery system, the ZnF16Pc generates reactive oxygen species (ROS) when it is exposed to light and will cause the immunogenic cell death (ICD) of the tumor cell, while at the same time, NLG919 will inhibit the activity of the immunosuppressive enzyme IDO. Research both in vitro and in vivo have shown that this treatment produced increased numbers of CD8^+^ T-Cell infiltration into the tumor, decreased numbers of regulatory T-cells (T-regs), and decreased numbers of Myeloid Derived Suppressor Cells (MDSCs) within the tumor. Approximately thirty percent (30%) of the animals in the study completely regressed their tumors and had an effective immune response when challenged with secondary tumors, resulting in a significant increase in both the suppression of tumor development and the survival of tumor bearing animals compared to control groups [173].

The Tumor Microenvironment (TME) consists of a highly complex system made up of tumor cells, infiltrating immune cells, fibroblasts, vascular endothelial cells, and other host cells, along with a multitude of bioactive molecules secreted by these host cells including cytokines, growth factors, and exosomes. Within the TME are high levels of interstitial fluid pressure (IFP) that limit the amount of drugs delivered to the tumor [174,175,176,177,178,179,180]. To develop a solution to this challenge, researchers have developed a synergistic strategy to modify TME resistance through the use of mild hyperthermia in combination with a smart drug delivery system (SDDS), thus increasing drug availability and therapeutic benefits in Triple Negative Breast Cancer (TNBC). The experiment utilized microwave (MW) irradiation to induce mild hyperthermia, activating the microwave sensitizer 1-butyl-3-methylimidazole-L-lactate (BML) to generate a synergistic cascade effect, thereby amplifying the hyperthermic effect and enabling real-time triggered precise release of paclitaxel (Ptx) at the tumor site. Microwave irradiation lead to significant anticancer efficacy of the combined treatment strategy, where tumor inhibition rates of up to 88% were achieved [181].

Low intensity focused ultrasound (LIFU) has been found to exhibit unique biological effects that are non-thermal in nature and that may have clinical application in the treatment of cancer. In particular, LIFU can alter the microenvironment of the tumor system and to enhance the therapeutic tolerance of cancer cells via non-thermal methods that include mechanical vibration, cavitation, and acoustic pore formation [182,183,184]. As an example, to address the short-comings of traditional treatments of retinoblastoma (RB), researchers developed a PLGA-Perfluorohexane-Fe_3_O_4_-GOx (PPFG) nanoparticle that can be triggered by LIFU. This nanoparticle system combines the unique properties of a nanozyme with chemodynamic therapy (CDT) to improve the efficacy of anticancer agents, while decreasing the associated side effects. Research further shows that the PPFG NPs, via the EPR effect, can be used to target and enrich in tumor tissue. Additionally, LIFU irradiation causes a phase change in the PFH core from liquid to gas, which enhances the release rate of anticancer agents. The released glucose oxidase (GOx) consumes glucose within tumor cells, inducing a tumor starvation effect; meanwhile, Fe_3_O_4_-mediated Fenton reaction generates a large amount of reactive oxygen species (ROS). These mechanisms ultimately achieve synergistic therapy for retinoblastoma [183].

**Figure 5:**
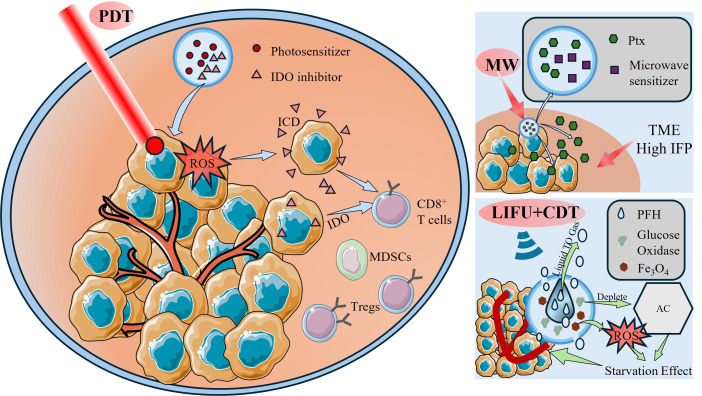
Schematic diagrams of various nanomedicine-based synergistic strategies for tumor microenvironment (TME) modulation and therapeutic efficacy enhancement. The blue arrow in the figure indicates that under PDT treatment, the photosensitizer generates reactive oxygen (ROS) species at the tumor site, activating CD8^+^ T cells and thereby triggering the immunogenic death of tumor cells. At the same time, the therapeutic drug inhibits ndoleamine 2,3-dioxygenase (IDO), reducing the immunosuppressive activity of Tregs and MDSCs. The red arrow indicates that there is a relatively high interstitial fluid pressure (IFP) within the TME, which restricts the delivery of drugs to the tumor. Enhancing microwave (MW) hyperthermia through the use of microwave sensitizers, thereby facilitating the release of paclitaxel or improving the therapeutic effect. The green arrow indicates that Fe_3_O_4_, under the excitation of LIFU, mediates a chemical kinetic reaction to generate ROS. Perfluorohexane (PFH) undergoes a phase change from liquid to gas state at the core under low intensity focused ultrasound (LIFU), thereby accelerating the release of anti-cancer drugs. The released glucose oxidase (GOx) can consume the glucose within tumor cells, resulting in a tumor starvation effect.

A schematic diagram of the mechanism of adjuvant combination therapy for tumors is shown in Figure 5.

## 5. Conclusions and Prospects

As previously mentioned, PLGA-based nanodrug delivery systems have demonstrated significant advantages in enhancing tumor immunotherapy and have garnered extensive attention and research over the past few decades. Their excellent biocompatibility and biodegradability have laid a solid theoretical and practical foundation for clinical translation. Diverse targeted modification strategies such as peptide, aptamer, and biomolecular-mediated approaches, along with adjuvant combination therapy modalities including photodynamic therapy, mild hyperthermia, and low-intensity focused ultrasound triggering techniques, have further significantly expanded their application prospects in the field of precision oncology. From a clinical translation perspective, however, most clinical studies on PLGA in the field of anti-tumor immunotherapy have only progressed to Phase I/II (e.g., NCT03066245, NCT05456022, NCT04619056), and several challenges remain to be addressed [185]. The primary challenge lies in significant barriers within the chemical, manufacturing, and control (CMC) domain. Currently, it remains challenging to achieve repeatable and large-scale synthesis of PLGA NPs with precise functionalization and active targeting properties, and completely eliminating batch-to-batch variations remains a major obstacle. Furthermore, if highly toxic chemotherapeutic agents undergo premature “burst release” during systemic circulation, they may pose non-targeted toxicity risks to NPs before reaching the tumor site [185,186,187,188,189]. For example, the PRECIOUS-01 trial is the first Phase I study of PLGA-based immunomodulatory nanomedicine delivering NY-ESO-1 and treitolceramide-6. Preclinical results showed good tolerance and immune activation. However, clinical translation faces challenges: NY-ESO-1 is not universally expressed, limiting its applicability, and its spatial heterogeneity in tumors may reduce efficacy. Additionally, the small sample size restricts evaluation mainly to safety rather than therapeutic effectiveness [189].

PLGA undergoes stepwise degradation via ester bond hydrolysis, generating lactate and glycolic acid monomers, which are ultimately metabolized through the tricarboxylic acid cycle into CO_2_ and H_2_O for excretion. However, the complex biological effects of PLGA degradation products (LA, GA) in TME remain incompletely elucidated, with different metabolic pathways potentially exerting dual impacts on immune responses. PLGA degradation products may further reduce the pH value of TME. Some studies have demonstrated that high concentrations of lactic acid and its associated acidic microenvironment directly inhibit the migration, infiltration, and cytotoxic function of CD8^+^ T cells, reduce the secretion of effector factors such as IFN-γ, and induce T cell exhaustion. Simultaneously, it promotes the proliferation of M2 macrophages, regulatory T cells, and myeloid-derived suppressor cells, thereby enhancing immunosuppression and promoting tumor growth [190,191,192]. However, existing research indicated that lactic acid can inhibit histone deacetylation, thereby upregulating the expression of the transcription factor TCF-1, which facilitates the expansion of stem cell-like CD8^+^ T cells and enhances their persistence and antitumor activity. Additionally, prolonged mild extracellular acidosis restricts methionine metabolism, maintains the dryness characteristics of T cells, improves mitochondrial function, and reduces cellular exhaustion. T cells pretreated with lactic acid exhibited superior tumor-suppressive effects after adoptive reinfusion [193,194,195,196]. Researchers should systematically investigate the in vivo metabolic kinetics and clearance pathways of PLGA and its degradation products, followed by optimization strategies such as surface functionalization, material composites modification, and microstructural regulation to enhance membrane materials and improve PLGA’s application potential in oncology therapy. Additionally, the preparation of PLGA NPs is influenced by multiple factors, including molecular weight, structural composition, solvent selection, and emulsification methods. Significant variations in particle size, morphology, and drug loading efficiency are observed among PLGA NPs prepared by different techniques. These differences not only directly affect nanoparticle behavior in vivo, such as passive targeting and enrichment efficiency at tumor sites, cellular internalization capacity, and drug-controlled release properties, but may also indirectly modulate immune cell functions in the tumor microenvironment. For instance, smaller PLGA NPs demonstrate enhanced penetration of tumor vascular endothelial spaces, while spherical particles exhibit superior macrophage phagocytosis and antigen presentation compared to irregularly shaped particles. Therefore, further research on PLGA’s in vivo metabolic mechanisms and preparation processes is essential for diverse tumor types and immunotherapy strategies.

Beyond tumor immunotherapy, PLGA is extensively utilized in other fields such as tissue engineering, chronic disease treatment, ophthalmic drug delivery, and medical imaging and diagnostics, owing to the inherent excellent properties and multifunctional applicability of PLGA materials. In tissue engineering, PLGA is often processed into porous scaffolds, whose controllable degradation rates and suitable mechanical properties make it a key material for bone, cartilage, and skin repair. For chronic disease treatment, PLGA NPs can encapsulate biomacromolecular drugs such as insulin and growth hormone, achieving sustained slow release and significantly reducing dosing frequency in patients with diabetes and growth hormone deficiency. In ophthalmology, PLGA carriers can prolong the retention time of anti-angiogenic drugs in the eye, enhancing therapeutic efficacy for ocular diseases such as macular degeneration. In medical imaging, PLGA serves as a carrier for contrast agents, loaded with fluorescent or radioactive probes to integrate disease diagnosis and treatment. In summary, PLGA holds broad application prospects in the medical field.

Furthermore, with the rapid advancement of artificial intelligence (AI) technology, future research may integrate single-cell sequencing techniques with AI to provide novel insights for personalized PLGA nanocarrier design. Specifically, this approach identifies highly expressed receptor targets based on patient-specific tumor gene expression profiles and customizes corresponding surface ligands, while optimizing the design of PLGA nanocarriers with AI assistance. Within the framework of AI and multimodal synergistic therapy strategies, this method holds promise for achieving personalized cancer treatment.

## Figures and Tables

**Table 1:** Clinical applications of PLGA materials with different LA:GA ratios.

LA:GA Ratio	Degradation Period	Typical Application Scenarios	Key Characteristics	Drug	Reference
50:50	1–2 months	Short-term implant; vaccine adjuvant	Fastest degradation, highest hydrophilicity	Lupron Depot^®^ (Leuprolide Sustained-Release Microspheres, Prostate Cancer/Endometriosis)	[21]
65:35	2–3 months	Bone repair scaffold; local anesthesia	Balanced degradation and mechanical properties	Arestin^®^ (Minocycline Periodontal Bag Sustained-Release Particles for Periodontitis Treatment)	[22]
75:25	3–4 months	Long-acting contraceptive; antitumor agents	A widely used standard model compatible with most hydrophobic drugs	Bydureon^®^ (Insulin Glargine Extended-Release Microspheres, Type 2 Diabetes)	[23]
85:15	4–6 months	Neural repair; gene vector	Slowest degradation, maintaining long-term sustained-release effect	Vivitrol^®^ (Naltrexone Sustained-Release Intramuscular Injection, Opioid/Alcohol Dependence)	[24]

**Table 2:** Synthesis Method and Drug Loading Strategy of PLGA Nanoparticles.

Preparation Method	Cargo Type	Typical Loading Method	Tumor Therapy Example	Reference
Solvent evaporation	Hydrophobic drugs (e.g., paclitaxel, doxorubicin base), small molecules	Dissolve drug and PLGA in organic solvent, emulsify in water, and evaporate solvent	Crocetin-loaded PLGA NPs; Noscapine-loaded PLGA NPs	[30,31,32]
Double emulsion	Hydrophilic (e.g., proteins, peptides, siRNA, DNA)	Dissolve drug in inner water phase, emulsify with PLGA organic phase, then disperse into outer water phase	Anti-CD47-loaded PLGA NPs; Capecitabine-loaded PLGA NPs	[33,34]
Nanoprecipitation	Hydrophobic or amphiphilic drugs	Dissolve PLGA and drug in water-miscible organic solvent and inject into aqueous phase for spontaneous NPs formation	Paclitaxel-loaded PLGA NPs; Docetaxel-loaded PLGA NPs	[35,36,37]
Spray drying	Thermostable drugs, small molecules, proteins, vaccines	PLGA and drug solution is atomized into microdroplets, with solvent rapidly evaporating in hot gas to form solid NPs	Sorafenib-loaded PLGA NPs; Simvastatin-loaded PLGA NPs	[38,39]
Microfluidic Control	Formulations requiring high homogeneity, small molecules, nucleic acids, proteins	Precise control of fluid behavior enables the controllable preparation and solidification molding of monodisperse microcarriers	5-fluorouracil-loaded PLGA NPs; Taxane-loaded PLGA NPs	[40,41]

**Table 3:** Cumulative release percentage of BSA-PLGA drug within 24 h.

Drug Delivery System	Environment Condition	Cumulative Release Rate (24 h)
PLGA NPs	pH 7.4	52 ± 2%
Non-crosslinked BSA-PLGA NPs	pH 7.4	24 ± 5%
crosslinked BSA-PLGA NPs	pH 7.4	Extremely low and negligible
crosslinked BSA-PLGA NPs	pH 6.5	Extremely low and negligible
crosslinked BSA-PLGA NPs	pH 5.8	45 ± 5%
crosslinked BSA-PLGA NPs	pH 5.0	79 ± 4%
crosslinked BSA-PLGA NPs	NO GSH	Extremely low and negligible
crosslinked BSA-PLGA NPs	0.5 mM GSH	18 ± 3%
crosslinked BSA-PLGA NPs	10.0 mM GSH	48 ± 3%

## Data Availability

No new data were created or analyzed in this study. Data sharing is not applicable to this article.

## References

[1] GBD 2023 Cancer Collaborators (2025). The Global, Regional, and National Burden of Cancer, 1990–2023, with Forecasts to 2050: A Systematic Analysis for the Global Burden of Disease Study 2023. Lancet.

[2] Xi J., Ji C., Sun H., Wu Y., Shi C., Li S., Yang T., Shen Y., Li Y., Fan Y. (2025). Research Progress on New Physical Therapies for Cancer (Review). Oncol. Lett..

[3] Zhang M., Liu C., Tu J., Tang M., Ashrafizadeh M., Nabavi N., Sethi G., Zhao P., Liu S. (2025). Advances in Cancer Immunotherapy: Historical Perspectives, Current Developments, and Future Directions. Mol. Cancer.

[4] Ye T., Li F., Ma G., Wei W. (2021). Enhancing Therapeutic Performance of Personalized Cancer Vaccine via Delivery Vectors. Adv. Drug Deliv. Rev..

[5] Su Y., Zhang B., Sun R., Liu W., Zhu Q., Zhang X., Wang R., Chen C. (2021). PLGA-Based Biodegradable Microspheres in Drug Delivery: Recent Advances in Research and Application. Drug Deliv..

[6] Rocha C.V., Gonçalves V., Da Silva M.C., Bañobre-López M., Gallo J. (2022). PLGA-Based Composites for Various Biomedical Applications. Int. J. Mol. Sci..

[7] Yang J., Zeng H., Luo Y., Chen Y., Wang M., Wu C., Hu P. (2024). Recent Applications of PLGA in Drug Delivery Systems. Polymers.

[8] Beach M.A., Nayanathara U., Gao Y., Zhang C., Xiong Y., Wang Y., Such G.K. (2024). Polymeric Nanoparticles for Drug Delivery. Chem. Rev..

[9] Wang J., Zhao W., Zhang Z., Liu X., Xie T., Wang L., Xue Y., Zhang Y. (2024). A Journey of Challenges and Victories: A Bibliometric Worldview of Nanomedicine since the 21st Century. Adv. Mater..

[10] Mitchell M.J., Billingsley M.M., Haley R.M., Wechsler M.E., Peppas N.A., Langer R. (2021). Engineering Precision Nanoparticles for Drug Delivery. Nat. Rev. Drug Discov..

[11] Zhang D., Liu L., Wang J., Zhang H., Zhang Z., Xing G., Wang X., Liu M. (2022). Drug-Loaded PEG-PLGA Nanoparticles for Cancer Treatment. Front. Pharmacol..

[12] Rezvantalab S., Drude N.I., Moraveji M.K., Güvener N., Koons E.K., Shi Y., Lammers T., Kiessling F. (2018). PLGA-Based Nanoparticles in Cancer Treatment. Front. Pharmacol..

[13] Narmani A., Jahedi R., Bakhshian-Dehkordi E., Ganji S., Nemati M., Ghahramani-Asl R., Moloudi K., Hosseini S.M., Bagheri H., Kesharwani P. (2023). Biomedical Applications of PLGA Nanoparticles in Nanomedicine: Advances in Drug Delivery Systems and Cancer Therapy. Expert. Opin. Drug Deliv..

[14] Wang R., Cheng L., He L., Du C., Wang H., Peng B., Yu X., Liu W., Luo W., Ran H. (2024). Nitric Oxide Nano-Reactor DNMF/PLGA Enables Tumor Vascular Microenvironment and Chemo-Hyperthermia Synergetic Therapy. J. Nanobiotechnol..

[15] Zhang Y., Dong Y., Fu H., Huang H., Wu Z., Zhao M., Yang X., Guo Q., Duan Y., Sun Y. (2021). Multifunctional Tumor-Targeted PLGA Nanoparticles Delivering Pt(IV)/siBIRC5 for US/MRI Imaging and Overcoming Ovarian Cancer Resistance. Biomaterials.

[16] Dong M., Liu Y., Xiao Y., Wu Q., Guan M., Xiao Z., Liu J., Cao L., Lu Y. (2025). Tumor-Targeted PLGA Nanospheres Enhance Therapeutic Effect of Lenvatinib in Hepatocellular Carcinoma via Photothermal and Photodynamic Therapy. ACS Appl. Mater. Interfaces.

[17] Kondiah P. (2020). The Design of Poly(Lactide-Co-Glycolide) Nanocarriers for Medical Applications. Front. Bioeng. Biotechnol..

[18] Elmowafy E.M., Tiboni M., Soliman M.E. (2019). Biocompatibility, Biodegradation and Biomedical Applications of Poly(Lactic Acid)/Poly(Lactic-Co-Glycolic Acid) Micro and Nanoparticles. J. Pharm. Investig..

[19] El-Hammadi M.M., Arias J.L. (2022). Recent Advances in the Surface Functionalization of PLGA-Based Nanomedicines. Nanomaterials.

[20] Roces C.B., Christensen D., Perrie Y. (2020). Translating the Fabrication of Protein-Loaded Poly(Lactic-Co-Glycolic Acid) Nanoparticles from Bench to Scale-Independent Production Using Microfluidics. Drug Deliv. Transl. Res..

[21] Zhou J., Hirota K., Ackermann R., Walker J., Wang Y., Choi S., Schwendeman A., Schwendeman S.P. (2018). Reverse Engineering the 1-Month Lupron Depot^®^. AAPS J..

[22] Li Z., Huang W., Zhang M., Huo Y., Li F., Song L., Wu S., Yang Q., Li X., Zhang J. (2024). Minocycline-Loaded nHAP/PLGA Microspheres for Prevention of Injury-Related Corneal Angiogenesis. J. Nanobiotechnol..

[23] White C., Schwendeman S.P. Formulation and Characterization of Exenatide-Loaded PLGA Microspheres Prepared by Coacervation—PubMed. https://pubmed.ncbi.nlm.nih.gov/41364402/.

[24] Hua Y., Wang Z., Wang D., Lin X., Liu B., Zhang H., Gao J., Zheng A. (2021). Key Factor Study for Generic Long-Acting PLGA Microspheres Based on a Reverse Engineering of Vivitrol^®^. Molecules.

[25] Grama C.N., Ankola D.D., Kumar M.N.V.R. (2011). Poly(Lactide-*Co*-Glycolide) Nanoparticles for Peroral Delivery of Bioactives. Curr. Opin. Colloid Interface Sci..

[26] Pourasghar M., Koenneke A., Meiers P., Schneider M. (2019). Development of a Fast and Precise Method for Simultaneous Quantification of the PLGA Monomers Lactic and Glycolic Acid by HPLC. J. Pharm. Anal..

[27] Li J., Tao R., Wu W., Cao H., Xin J., Li J., Guo J., Jiang L., Gao C., Demetriou A.A. (2010). 3D PLGA Scaffolds Improve Differentiation and Function of Bone Marrow Mesenchymal Stem Cell–Derived Hepatocytes. Stem Cells Dev..

[28] Gong M., Zha Y., Lu S., Zhang T., Wang X., Liu L., Fan C., Zeng H., Wang D., Song T. (2026). Efficacy and Safety of the Low-Temperature-Derived 3D Printed Biodegradable Mg-Containing Composite Porous Scaffold for Bone Defect Repair: A Prospective and Multi-Center Randomized Controlled Trial. Biomaterials.

[29] Horvath D., Basler M. (2023). PLGA Particles in Immunotherapy. Pharmaceutics.

[30] Hafezi Ghahestani Z., Alebooye Langroodi F., Mokhtarzadeh A., Ramezani M., Hashemi M. (2017). Evaluation of Anti-Cancer Activity of PLGA Nanoparticles Containing Crocetin. Artif. Cells Nanomed. Biotechnol..

[31] Yadav K., Yadav D., Yadav M., Kumar S. (2015). Noscapine Loaded PLGA Nanoparticles Prepared Using Oil-in-Water Emulsion Solvent Evaporation Method. J. Nanopharm. Drug Deliv..

[32] Chen X., Li Y., Zhang Y., Li G. (2019). Formulation, Characterization and Evaluation of Curcumin- Loaded PLGA- TPGS Nanoparticles for Liver Cancer Treatment. Drug Des. Devel Ther..

[33] Safari H., Felder M.L., Kaczorowski N., Eniola-Adefeso O. (2022). Effect of the Emulsion Solvent Evaporation Technique Cosolvent Choice on the Loading Efficiency and Release Profile of Anti-CD47 from PLGA Nanospheres. J. Pharm. Sci..

[34] Vedpathak S.K., Chivate N.D., Chivate A.N., Khanwelkar C.C. (2026). Development of Capecitabine-Encapsulated PEG-PLGA Nanoparticles Using Double Emulsion Solvent Evaporation for Sustained Drug Release. J. Pharm. Innov..

[35] Ghaly H.S.A., Seyedasli N., Varamini P. (2025). Enhanced Nanoprecipitation Method for the Production of PLGA Nanoparticles for Oncology Applications. AAPS J..

[36] Shi W., Zhang Z., Yuan Y., Xing E., Qin Y., Peng Z., Zhang Z., Yang K. (2013). Optimization of Parameters for Preparation of Docetaxel-Loaded PLGA Nanoparticles by Nanoprecipitation Method. J. Huazhong Univ. Sci. Technol. [Med. Sci.].

[37] Alshawsh D.M.A. (2017). Technical Aspects of Preparing PEG-PLGA Nanoparticles as Carrier for Chemotherapeutic agents by Nanoprecipitation Method. Int. J. Pharm..

[38] Scialabba C., Corsaro F., Drago S.E., Craparo E.F., Cavallaro G. (2025). Exploring a Mucoacting Spray-Dried Nanoparticle-in-Microparticle Inhalable Formulation for Targeted Pulmonary Delivery of Sorafenib in Lung Cancer Therapy. Int. J. Pharm..

[39] Anzar N., Mirza M.A., Anwer K., Khuroo T., Alshetaili A.S., Alshahrani S.M., Meena J., Hasan N., Talegaonkar S., Panda A.K. (2018). Preparation, Evaluation and Pharmacokinetic Studies of Spray Dried PLGA Polymeric Submicron Particles of Simvastatin for the Effective Treatment of Breast Cancer. J. Mol. Liq..

[40] Turkmen Koc S.N., Conger E., Ozturk S., Eroglu I., Ulubayram K. (2024). Production of 5-Fluorouracil-Loaded PLGA Nanoparticles with Toroidal Microfluidic System and Optimization of Process Variables by Design of Experiments. Int. J. Pharm..

[41] Bao Y., Maeki M., Ishida A., Tani H., Tokeshi M. (2022). Effect of Organic Solvents on a Production of PLGA-Based Drug-Loaded Nanoparticles Using a Microfluidic Device. ACS Omega.

[42] Yoo H.S., Lee K.H., Oh J.E., Park T.-G. (2000). In Vitro and in Vivo Anti-Tumor Activities of Nanoparticles Based on Doxorubicin-PLGA Conjugates. J. Control. Release.

[43] Zhao J., Weng G., Li J., Zhu J., Zhao J. (2018). Polyester-Based Nanoparticles for Nucleic Acid Delivery. Mater. Sci. Eng. C Mater. Biol. Appl..

[44] Wischke C., Schwendeman S.P. (2008). Principles of Encapsulating Hydrophobic Drugs in PLA/PLGA Microparticles. Int. J. Pharm..

[45] Ding D., Zhu Q. (2018). Recent Advances of PLGA Micro/Nanoparticles for the Delivery of Biomacromolecular Therapeutics. Mater. Sci. Eng. C Mater. Biol. Appl..

[46] Bose R.J., Lee S.-H., Park H. (2016). Lipid-Based Surface Engineering of PLGA Nanoparticles for Drug and Gene Delivery Applications. Biomater. Res..

[47] Laxane N., Yadav K.S. (2025). Efficient Co-Loading of Paclitaxel and Gefitinib in PLGA Nanoparticles: Use of Design of Experiments (DoE) Approach for Enhanced Efficacy Against MCF-7/MCF-7ADR Cells. Polym. Adv. Technol..

[48] Zheng Z., Wu Y., Ahmad A., Ramzan N., Kayitmazer A.B., Zhou X., Xu Y. (2024). Lyophilizable Polymer-Lipid Hybrid Nanoparticles with High Paclitaxel Loading. ACS Appl. Nano Mater..

[49] Raposo C.D., Costa R., Petrova K.T., Brito C., Scotti M.T., Cardoso M.M. (2020). Development of Novel Galactosylated PLGA Nanoparticles for Hepatocyte Targeting Using Molecular Modelling. Polymers.

[50] Vallorz E.L., Encinas-Basurto D., Schnellmann R.G., Mansour H.M. (2022). Design, Development, Physicochemical Characterization, and In Vitro Drug Release of Formoterol PEGylated PLGA Polymeric Nanoparticles. Pharmaceutics.

[51] Liu G., McEnnis K. (2022). Glass Transition Temperature of PLGA Particles and the Influence on Drug Delivery Applications. Polymers.

[52] Zeb A., Gul M., Nguyen T.T.L., Maeng H.J. (2022). Controlled Release and Targeted Drug Delivery with Poly(Lactic-Co-Glycolic Acid) Nanoparticles: Reviewing Two Decades of Research. J. Pharm. Investig..

[53] Guo T., Luo L., Wang L., Zhang F., Liu Y., Leng J. (2025). Smart Polymer Microspheres: Preparation, Microstructures, Stimuli-Responsive Properties, and Applications. ACS Nano.

[54] Sokolovskaya E., Rahmani S., Misra A.C., Bräse S., Lahann J. (2015). Dual-Stimuli-Responsive Microparticles. ACS Appl. Mater. Interfaces.

[55] Zhang M., Hu W., Cai C., Wu Y., Li J., Dong S. (2022). Advanced Application of Stimuli-Responsive Drug Delivery System for Inflammatory Arthritis Treatment. Mater. Today Bio.

[56] Zhang J., Wu G., Bobrin V.A. (2025). Nanocarrier Strategies for Deep Tumour Penetration. RSC Appl. Polym..

[57] Palanikumar L., Al-Hosani S., Kalmouni M., Nguyen V.P., Ali L., Pasricha R., Barrera F.N., Magzoub M. (2020). pH-Responsive High Stability Polymeric Nanoparticles for Targeted Delivery of Anticancer Therapeutics. Commun. Biol..

[58] Cespi M., Bonacucina G., Tiboni M., Casettari L., Cambriani A., Fini F., Perinelli D.R., Palmieri G.F. (2021). Insights in the Rheological Properties of PLGA-PEG-PLGA Aqueous Dispersions: Structural Properties and Temperature-Dependent Behaviour. Polymer.

[59] Qiao M., Chen D., Ma X., Liu Y. (2005). Injectable Biodegradable Temperature-Responsive PLGA-PEG-PLGA Copolymers: Synthesis and Effect of Copolymer Composition on the Drug Release from the Copolymer-Based Hydrogels. Int. J. Pharm..

[60] Zhang C., Wang X., Wang J., Qiu Y., Qi Z., Song D., Wang M. (2021). TCPP-Isoliensinine Nanoparticles for Mild-Temperature Photothermal Therapy. Int. J. Nanomed..

[61] Day N.B., Wixson W.C., Shields C.W. (2021). Magnetic Systems for Cancer Immunotherapy. Acta Pharm. Sin. B.

[62] Chen H.-A., Ma Y.-H., Hsu T.-Y., Chen J.-P. (2020). Preparation of Peptide and Recombinant Tissue Plasminogen Activator Conjugated Poly(Lactic-Co-Glycolic Acid) (PLGA) Magnetic Nanoparticles for Dual Targeted Thrombolytic Therapy. Int. J. Mol. Sci..

[63] Saengruengrit C., Ritprajak P., Wanichwecharungruang S., Sharma A., Salvan G., Zahn D.R.T., Insin N. (2018). The Combined Magnetic Field and Iron Oxide-PLGA Composite Particles: Effective Protein Antigen Delivery and Immune Stimulation in Dendritic Cells. J. Colloid Interface Sci..

[64] Hu Q., Katti P.S., Gu Z. (2014). Enzyme-Responsive Nanomaterials for Controlled Drug Delivery. Nanoscale.

[65] Guo C., Zeng Y., Ou Y., Gu Z., Luo K. (2025). Cathepsin B-Responsive Dendronized-Hyaluronic Acid Nanomedicine for Simultaneous Cancer Theranostics. MedComm–Oncology.

[66] Subhan M.A., Parveen F., Filipczak N., Yalamarty S.S.K., Torchilin V.P. (2023). Approaches to Improve EPR-Based Drug Delivery for Cancer Therapy and Diagnosis. J. Pers. Med..

[67] Acharya S., Sahoo S.K. (2011). PLGA Nanoparticles Containing Various Anticancer Agents and Tumour Delivery by EPR Effect. Adv. Drug Deliv. Rev..

[68] Maeda H., Wu J., Sawa T., Matsumura Y., Hori K. (2000). Tumor Vascular Permeability and the EPR Effect in Macromolecular Therapeutics: A Review. J. Control. Release.

[69] Maeda H. (2015). Toward a Full Understanding of the EPR Effect in Primary and Metastatic Tumors as Well as Issues Related to Its Heterogeneity. Adv. Drug Deliv. Rev..

[70] Mozar F.S., Chowdhury E.H. (2018). Impact of PEGylated Nanoparticles on Tumor Targeted Drug Delivery. Curr. Pharm. Design.

[71] Morshedi B., Esfandyari-Manesh M., Ghahremani M.H., Fatahi Y., Dinarvand R. (2025). Localized Co-Delivery of in-Situ Hydrogel Containing Ibrutinib-PLGA-PEG-Folate Nanoparticle and Octreotide Microsphere to Glioblastoma. Drug Deliv. Transl. Res..

[72] He C., Hu Y., Yin L., Tang C., Yin C. (2010). Effects of Particle Size and Surface Charge on Cellular Uptake and Biodistribution of Polymeric Nanoparticles. Biomaterials.

[73] Sharifi M., Cho W.C., Ansariesfahani A., Tarharoudi R., Malekisarvar H., Sari S., Bloukh S.H., Edis Z., Amin M., Gleghorn J.P. (2022). An Updated Review on EPR-Based Solid Tumor Targeting Nanocarriers for Cancer Treatment. Cancers.

[74] Colby A.H., Kirsch J., Patwa A.N., Liu R., Hollister B., McCulloch W., Burdette J.E., Pearce C.J., Oberliels N.H., Colson Y.L. (2023). Radiolabeled Biodistribution of Expansile Nanoparticles: Intraperitoneal Administration Results in Tumor Specific Accumulation. ACS Nano.

[75] Khan M.S., Rehman U., Alqahtani T., Al Shmrany H., Gupta G., Goh K.W., Sahebkar A., Kesharwani P. (2025). Advances in PLGA-Based Polymeric Nanocarriers for Colorectal Cancer Therapy: Overcoming Chemoresistance through Controlled Delivery Strategies. Mol. Cancer.

[76] Nguyen L.N.M., Ngo W., Lin Z.P., Sindhwani S., MacMillan P., Mladjenovic S.M., Chan W.C.W. (2024). The Mechanisms of Nanoparticle Delivery to Solid Tumours. Nat. Rev. Bioeng..

[77] Yuan L., Chen Q., Riviere J.E., Lin Z. (2023). Pharmacokinetics and Tumor Delivery of Nanoparticles. J. Drug Deliv. Sci. Technol..

[78] Ejigah V., Owoseni O., Bataille-Backer P., Ogundipe O.D., Fisusi F.A., Adesina S.K. (2022). Approaches to Improve Macromolecule and Nanoparticle Accumulation in the Tumor Microenvironment by the Enhanced Permeability and Retention Effect. Polymers.

[79] Cheng H., Liao J., Ma Y., Sarwar M.T., Yang H. (2025). Advances in Targeted Therapy for Tumor with Nanocarriers: A Review. Mater. Today Bio.

[80] Ma J., Yin W., Zhang X., Lin L., Bao Y., Dai L., Cao H., Chen H., Yu J., Yang J. (2024). Enhanced EPR Effects by Tumour Stromal Cell Mimicking Nanoplatform on Invasive Pituitary Adenoma. Mater. Today Bio.

[81] Attia M.F., Anton N., Wallyn J., Omran Z., Vandamme T.F. (2019). An Overview of Active and Passive Targeting Strategies to Improve the Nanocarriers Efficiency to Tumour Sites. J. Pharm. Pharmacol..

[82] Khan I., Gothwal A., Sharma A.K., Kesharwani P., Gupta L., Iyer A.K., Gupta U. PLGA Nanoparticles and Their Versatile Role in Anticancer Drug Delivery—Critical Reviews^TM^ in Therapeutic Drug Carrier Systems, Volume 33, 2016, Issue 2—Begell House Digital Library. https://www.dl.begellhouse.com/journals/3667c4ae6e8fd136,3e868ea50400cfbb,356e2ffb0a73d4f1.html.

[83] Zhong Y., Meng F., Deng C., Zhong Z. (2014). Ligand-Directed Active Tumor-Targeting Polymeric Nanoparticles for Cancer Chemotherapy. Biomacromolecules.

[84] Chiu H.I., Samad N.A., Fang L., Lim V. (2021). Cytotoxicity of Targeted PLGA Nanoparticles: A Systematic Review. RSC Adv..

[85] Sadat Tabatabaei Mirakabad F., Nejati-Koshki K., Akbarzadeh A., Yamchi M.R., Milani M., Zarghami N., Zeighamian V., Rahimzadeh A., Alimohammadi S., Hanifehpour Y. (2014). PLGA-Based Nanoparticles as Cancer Drug Delivery Systems. Asian Pac. J. Cancer Prev..

[86] Yang M., Li J., Gu P., Fan X. (2021). The Application of Nanoparticles in Cancer Immunotherapy: Targeting Tumor Microenvironment. Bioact. Mater..

[87] Khristenko N.A., Nagornov K.O., Garcia C., Gasilova N., Gant M., Druart K., Kozhinov A.N., Menin L., Chamot-Rooke J., Tsybin Y. (2025). Top-Down and Middle-Down Mass Spectrometry of Antibodies. Mol. Cell. Proteom..

[88] Kholodenko R.V., Kalinovsky D.V., Doronin I.I., Ponomarev E.D., Kholodenko I.V. (2019). Antibody Fragments as Potential Biopharmaceuticals for Cancer Therapy: Success and Limitations. Curr. Med. Chem..

[89] Chen W., Yuan Y., Jiang X. (2020). Antibody and Antibody Fragments for Cancer Immunotherapy. J. Control. Release.

[90] Jin Y., Zakeri S.E., Bahal R., Wiemer A.J. (2022). New Technologies Bloom Together for Bettering Cancer Drug Conjugates. Pharmacol. Rev..

[91] Wang T., Liu L., Voglmeir J. mAbs N-Glycosylation: Implications for Biotechnology and Analytics-Web of Science Core Collection. https://webofscience.clarivate.cn/wos/woscc/full-record/WOS:000804197400007.

[92] Ghirardello M., Shyam R., Liu X., Garcia-Millan T., Sittel I., Ramos-Soriano F.J., Kurian K., Galan M.C. (2022). Carbon Dot-Based Fluorescent Antibody Nanoprobes as Brain Tumour Glioblastoma Diagnostics. Nanoscale Adv..

[93] Wang L., Bi S., Li Z., Liao A., Li Y., Yang L., Zhou X., Gao Y., Liu X., Zou Y. (2025). Napabucasin Deactivates STAT3 and Promotes Mitoxantrone-Mediated cGAS-STING Activation for Hepatocellular Carcinoma Chemo-Immunotherapy. Biomaterials.

[94] Giglio A., Chiesa E., Iamele L., Rubes D., Serra M., Riva F., Dorati R., Conti B., de Jonge H., Genta I. (2025). Engineering Anti-c-MET scFv-Conjugated PLGA Nanoparticles for Precision Verteporfin Delivery in Lung Cancer Cells: A Formulation Study. Int. J. Pharm..

[95] Chapman A.P. (2002). PEGylated Antibodies and Antibody Fragments for Improved Therapy: A Review. Adv. Drug Deliv. Rev..

[96] Castro A., Pittini Á., Berois N., Faccio R., Miranda P., Mombrú Á.W., Osinaga E., Pardo H. Development, Characterization, and Evaluation of Chi-Tn mAb-Functionalize1d DOTAP-PLGA Hybrid Nanoparticles Loaded with Docetaxel for Lung Cancer Therapy-Web of Science Core Collection. https://webofscience.clarivate.cn/wos/woscc/full-record/WOS:001429759600001.

[97] Xiao W., Jiang W., Chen Z., Huang Y., Mao J., Zheng W., Hu Y., Shi J. (2025). Advance in Peptide-Based Drug Development: Delivery Platforms, Therapeutics and Vaccines. Signal Transduct. Target. Ther..

[98] Guzelsoy G., Elorza S.D., Ros M., Schachtner L.T., Hayashi M., Hobson-Gutierrez S., Rundstrom P., Brunner J.S., Pillai R., Walkowicz W.E. (2025). Cooperative Nutrient Scavenging Is an Evolutionary Advantage in Cancer. Nature.

[99] Maksymova L., Pilger Y.A., Nuhn L., Van Ginderachter J.A. (2025). Nanobodies Targeting the Tumor Microenvironment and Their Formulation as Nanomedicines. Mol. Cancer.

[100] Tao Z., Li Y., Huang Y., Hu L., Wang S., Wan L., She T., Shi Q., Lu S., Wang X. (2025). Multivalent Assembly of Nucleolin-Targeted F3 Peptide Potentiates TRAIL’s Tumor Penetration and Antitumor Effects. J. Control. Release.

[101] Xiao Y., He Z., Li W., Chen D., Niu X., Yang X., Zeng W., Wang M., Qian Y., Su Y. (2025). A Covalent Peptide-Based Lysosome-Targeting Protein Degradation Platform for Cancer Immunotherapy. Nat. Commun..

[102] Han H.-L., Su J.-Y., Zhao X.-H., Hou D.-D., Li Y.-M. (2025). Peptide-Based Strategies in PLGA-Enhanced Tumor Therapy. J. Pept. Sci..

[103] Butreddy A., Gaddam R.P., Kommineni N., Dudhipala N., Voshavar C. (2021). PLGA/PLA-Based Long-Acting Injectable Depot Microspheres in Clinical Use: Production and Characterization Overview for Protein/Peptide Delivery. Int. J. Mol. Sci..

[104] Zhang C., Yang L., Wan F., Bera H., Cun D., Rantanen J., Yang M. (2020). Quality by Design Thinking in the Development of Long-Acting Injectable PLGA/PLA-Based Microspheres for Peptide and Protein Drug Delivery. Int. J. Pharm..

[105] Allahyari M., Mohit E. (2016). Peptide/Protein Vaccine Delivery System Based on PLGA Particles. Hum. Vaccines Immunother..

[106] Cheng T.-M., Chang W.-J., Chu H.-Y., De Luca R., Pedersen J.Z., Incerpi S., Li Z.-L., Shih Y.-J., Lin H.-Y., Wang K. (2021). Nano-Strategies Targeting the Integrin Avβ3 Network for Cancer Therapy. Cells.

[107] Wang C., Su L., Wu C., Wu J., Zhu C., Yuan G. (2016). RGD Peptide Targeted Lipid-Coated Nanoparticles for Combinatorial Delivery of Sorafenib and Quercetin against Hepatocellular Carcinoma. Drug Dev. Ind. Pharm..

[108] Shin Y.C., Kim J., Kim S.E., Song S.-J., Hong S.W., Oh J.-W., Lee J., Park J.-C., Hyon S.-H., Han D.-W. (2017). RGD Peptide and Graphene Oxide Co-Functionalized PLGA Nanofiber Scaffolds for Vascular Tissue Engineering. Regen. Biomater..

[109] Yadav B., Chauhan M., Shekhar S., Kumar A., Mehata A.K., Nayak A.K., Dutt R., Garg V., Kailashiya V., Muthu M.S. (2023). RGD-Decorated PLGA Nanoparticles Improved Effectiveness and Safety of Cisplatin for Lung Cancer Therapy. Int. J. Pharm..

[110] Chauhan M., Sonali, Shekhar S., Yadav B., Garg V., Dutt R., Mehata A.K., Goswami P., Koch B., Muthu M.S. (2024). AS1411 Aptamer/RGD Dual Functionalized Theranostic Chitosan-PLGA Nanoparticles for Brain Cancer Treatment and Imaging. Biomater. Adv..

[111] Lin C.-Y., Fang J.-Y., Hsiao C.-Y., Lee C.-W., Alshetaili A., Lin Z.-C. (2025). Dual Cell-Penetrating Peptide-Conjugated Polymeric Nanocarriers for miRNA-205–5p Delivery in Gene Therapy of Cutaneous Squamous Cell Carcinoma. Acta Biomater..

[112] Pyla M., Kankipati S., Sumithra B., Mishra P.K., Mishra B., Mandal S.K., Panda J., Chopra H., Avula S.K., Attia M.S. (2025). Bacterial Proteins and Peptides as Potential Anticancer Agents: A Novel Search for Protein-Based Therapeutics. Curr. Med. Chem..

[113] Rai N., Tiwari R.T., Sahu A., Verma E., Rathore S., Patil S., Patil A.G. (2025). Exploring Tryptophan-Based Short Peptides: Promising Candidate for Anticancer and Antimicrobial Therapies. Anti-Cancer Agents Med. Chem..

[114] Fernandes S., Cavalieri F. (2025). Antimicrobial Peptides Boosted by Ultrasound. Nat. Biomed. Eng..

[115] Ladaycia A., Lemaire L., Pailhoriès H., Lautram N., Franconi F., Pigeon P., Jaouen G., Passirani C., Lepeltier E. (2026). Self-Assemblies from Prodrugs Composed of Antimicrobial Peptides: A Revolution in Local Lung Cancer Treatment, with Microbiota as a Main Actor. Drug Deliv. Transl. Res..

[116] Sakhawat A., Khan M.U., Khan S., Ahmed N., Alhegaili A.S., Alotaibi B.S. (2025). PD-L1 Targeting in Triple Negative Breast Cancer: In Silico and in Vitro Validation of Wasp Venom Peptide MP-1. Med. Oncol..

[117] Li Y., Zamay T.N., Luzan N.A., Pryakhin E.A., Styazhkina E.V., Osminkina L.A., Kolovskaya O.S., Dymova M.A., Kuligina E.V., Richter V.A. (2025). Aptamers as a New Frontier in Targeted Cancer Therapy. Adv. Drug Deliv. Rev..

[118] Egbe Vydaline A.O., Rozhkov S., Sosa G., Mallikaratchy P. (2025). Aptamers as Target-Specific Recognition Elements in Drug Delivery. Adv. Drug Deliv. Rev..

[119] Zhou H., Li Y., Wu W. (2024). Aptamers: Promising Reagents in Biomedicine Application. Adv. Biol..

[120] Driscoll J., Gondaliya P., Zinn D.A., Jain R., Yan I.K., Dong H., Patel T. (2025). Using Aptamers for Targeted Delivery of RNA Therapies. Mol. Ther..

[121] Shigdar S., Schrand B., Giangrande P.H., de Franciscis V. (2021). Aptamers: Cutting Edge of Cancer Therapies. Mol. Ther..

[122] Benedetto G., Fowler A., Langdon D., Raine M., White M.L., Ogle J., Garmon C., Ogle C., Richardson C. (2025). Aptamer-Coated PLGA Nanoparticles Selectively Internalize into Epithelial Ovarian Cancer Cells In Vitro and In Vivo. Biomolecules.

[123] Liu X., Sun M., Dai L., Li Y., Song R., Bian H., Zhang H. (2025). Functional Nucleic Acid-Powered Hybrid Nanocarriers for Synchronized Targeted Delivery of Dual-Solubility Therapeutics. J. Biotechnol..

[124] Zhang Y., Li W., Chen S., Zhang Y., Zhu Y., Lan F., Du H., Fan R., Zhu J., Pan W. (2025). Layered-Responsive Multivalent Tetrahedral DNA Framework-Decorated CRISPR-Cas12a Nanocapsule Enables Precise and Enhanced Tumor Chemotherapy. ACS Nano.

[125] Alsharif S.T., Gardouh A.M., Mandour M.F., Alaqais Z.M., Alharbi L.K., Almarwani M.J., Mokhtar H.I., Hisham F.A., Abdellah M.M., Mohamed G.M. (2024). Antitumor Activity and Targeting P53-PUMA mRNA Expression by 5-Flurouracil PLGA-Lipid Polymeric Nanoparticles in Mouse Mammary Carcinomas: Comparison to Free 5-Flurouracil. Toxicol. Mech. Methods.

[126] Zhang Z., Fan Y., Jiang S., Ma Y., Yu Y., Qing Y., Li Q., Liu Y., Shen S., Wang J. (2025). Recent Advances in mRNA Delivery Systems for Cancer Therapy. Adv. Sci..

[127] Agbaria M., Jbara-Agbaria D., Golomb G. (2025). Targeting siRNA Nanoparticles with ApoB Peptide: Formulation, Biodistribution, and Bioactivity in Pancreatic Tumor-Bearing Mice. J. Drug Deliv. Sci. Technol..

[128] Uti D.E., Omang W.A., Alum E.U., Ugwu O.P.-C., Wokoma M.A., Oplekwu R.I., Atangwho I.J., Egbung G.E. (2026). Targeting CD44-Hyaluronic Acid Signalling in Obesity Treatment: Insights from Small Molecules and Nanobioconjugates. Nutr. Metab. Insights.

[129] Goren A., Bao Z., Martinez Lozano J.P. (2025). A Formulation Dataset of Poly(Lactide-Co-Glycolide) Nanoparticles for Small Molecule Delivery. Sci. Data.

[130] Erdag D., Garrido M.D., Basoglu H., Yazgan I., Amoros P., Yalcintepe L., Toprak M.S. (2025). Cold Plasma Triggered Cell Death with a Curcumin and Capecitabine Loaded Magnetic Nanocluster-Based Multifunctional System on the MCF-7 Cell Line: A Smart Therapy Platform. J. Mat. Chem. B.

[131] Dual Targeting of FR+CD44 Overexpressing Tumors by Self-Assembled Nanoparticles Quantitatively Conjugating Folic Acid-Hyaluronic Acid to the GSH-Sensitively Modified Podophyllotoxin-Web of Science Core Collection. https://webofscience.clarivate.cn/wos/woscc/full-record/WOS:001399224200001.

[132] Plant Galactolipid dLGG Suppresses Lung Metastasis of Melanoma through Deregulating TNF-α-Mediated Pulmonary Vascular Permeability and Circulating Oxylipin Dynamics in Mice-Web of Science Core Collection. https://webofscience.clarivate.cn/wos/woscc/full-record/WOS:000451115900019.

[133] Rahbar K., Giesel F.L., Herrmann K., Yun M., Watabe T., Rudolph I., Hoepping A., Maurer T. (2026). Efficacy of [18F]PSMA-1007 PET/CT in Primary Staging of Prostate Carcinoma: A Systematic Review and Metaanalysis. J. Nucl. Med..

[134] Narmani A., Ganji S., Amirishoar M., Jahedi R., Kharazmi M.S., Jafari S.M. (2023). Smart Chitosan-PLGA Nanocarriers Functionalized with Surface Folic Acid Ligands against Lung Cancer Cells. Int. J. Biol. Macromol..

[135] Morshedi B., Esfandyari-Manesh M., Atyabi F., Ghahremani M.H., Dinarvand R. (2025). Local Delivery of Ibrutinib by Folate Receptor-Mediated Targeting PLGA-PEG Nanoparticles to Glioblastoma Multiform: In Vitro and in Vivo Studies. J. Drug Target..

[136] Son J., Yang S.M., Yi G., Roh Y.J., Park H., Park J.M., Choi M.-G., Koo H. (2018). Folate-Modified PLGA Nanoparticles for Tumor-Targeted Delivery of Pheophorbide a In Vivo. Biochem. Biophys. Res. Commun..

[137] Tiwari G., Panda S., Diyya A.S.M., Thomas N.V., Deka T., Rudrangi S.R.S., Patel G., Sharma P. (2025). Design and Optimization of PLGA-Based Gemcitabine Nanocapsule for Enhanced Pancreatic Cancer Efficacy. Investig. New Drugs.

[138] Pang L., Huang X., Zhu L., Xiao H., Li M., Guan H., Gao J., Jin H. (2022). Targeted killing of CD133^+^ lung cancer stem cells using paclitaxel-loaded PLGA-PEG nanoparticles with CD133 aptamers. J. South. Med. Univ..

[139] Bhattacharya S. (2021). Anti-EGFR-mAb and 5-Fluorouracil Conjugated Polymeric Nanoparticles for Colorectal Cancer. Recent Pat. Anti-Cancer Drug Discov..

[140] Sousa F., Lee H., Almeida M., Bazzoni A., Rothen-Rutishauser B., Petri-Fink A. (2024). Immunostimulatory Nanoparticles Delivering Cytokines as a Novel Cancer Nanoadjuvant to Empower Glioblastoma Immunotherapy. Drug Deliv. Transl. Res..

[141] Wu J., Wang X., Wang Y., Xun Z., Li S. (2024). Application of PLGA in Tumor Immunotherapy. Polymers.

[142] Yilma A.N., Sahu R., Subbarayan P., Villinger F., Coats M.T., Singh S.R., Dennis V.A. (2024). PLGA-Chitosan Encapsulated IL-10 Nanoparticles Modulate Chlamydia Inflammation in Mice. Int. J. Nanomed..

[143] Mihalik N.E., Wen S., Driesschaert B., Eubank T.D. (2021). Formulation and in Vitro Characterization of PLGA/PLGA-PEG Nanoparticles Loaded with Murine Granulocyte-Macrophage Colony-Stimulating Factor. AAPS PharmSciTech.

[144] Sun R., Chen Y., Pei Y., Wang W., Zhu Z., Zheng Z., Yang L., Sun L. (2024). The Drug Release of PLGA-Based Nanoparticles and Their Application in Treatment of Gastrointestinal Cancers. Heliyon.

[145] Rout S.R., Kenguva G., Sharma D., Sahebkar A., Aeri V., Kesharwani P., Dandela R. (2023). Gene Therapy Using PLGA Nanoparticles. Poly(lactic-Co-Glycolic Acid) (PLGA) Nanoparticles for Drug Delivery.

[146] Tunable Rigidity of PLGA Shell-Lipid Core Nanoparticles for Enhanced Pulmonary siRNA Delivery in 2D and 3D Lung Cancer Cell Models—PubMed. https://pubmed.ncbi.nlm.nih.gov/38237688/.

[147] Cai H., Li X., Liu Y., Ke J., Liu K., Xie Y., Xie C., Zhou D., Han M., Ji B. (2024). Decitabine-Based Nanoparticles for Enhanced Immunotherapy of Hepatocellular Carcinoma via DNA Hypermethylation Reversal. Chem. Eng. J..

[148] Preparation and Characterization of PLGA-Based Magnetic Polymer Nanoparticles for Targeting Pancreatic Adenocarcinoma. https://chinesesites.library.ingentaconnect.com/content/ben/cpd/2023/00000029/00000009/art00005.

[149] Alkilany A.M., Rachid O., Alkawareek M.Y., Billa N., Daou A., Murphy C.J. (2022). PLGA-Gold Nanocomposite: Preparation and Biomedical Applications. Pharmaceutics.

[150] Malinovskaya J., Salami R., Valikhov M., Vadekhina V., Semyonkin A., Semkina A., Abakumov M., Harel Y., Levy E., Levin T. (2022). Supermagnetic Human Serum Albumin (HSA) Nanoparticles and PLGA-Based Doxorubicin Nanoformulation: A Duet for Selective Nanotherapy. Int. J. Mol. Sci..

[151] Ji J., Qin H., Yang Y., Wu J., Wu J. (2022). The Targeting Imaging and Treatment Capacity of Gelsolin-Targeted and Paclitaxel-Loaded PLGA Nanoparticles in Vitro and in Vivo. Front. Bioeng. Biotechnol..

[152] Zhou Y., Wei R., Wang L., Li J., Wang W., Jiang G., Tan S., Li F., Wang X., Ma X. (2024). Tumor Targeting Peptide TMTP1 Modified Antigen Capture Nano-Vaccine Combined with Chemotherapy and PD-L1 Blockade Effectively Inhibits Growth of Ovarian Cancer. J. Nanobiotechnol..

[153] Cheng M., Chai Y., Rong G., Xin C., Gu L., Zhou X., Hong J. (2025). Nanotechnology-Based Strategies for Vaccine Development: Accelerating Innovation and Delivery. Biomater. Transl..

[154] Yu P., Jin Z., Meng L., Shi Z., Li M., Luo J., Zhu X., Yang L., Yin Y., Zhang C. (2026). Biomimetic Vesicles Engineered from Modified Tumour Cells Act as Personalized Vaccines for Post-Surgical Cancer Immunotherapy. Nat. Nanotechnol..

[155] Liu T., Yao W., Sun W., Yuan Y., Liu C., Liu X., Wang X., Jiang H. (2024). Components, Formulations, Deliveries, and Combinations of Tumor Vaccines. ACS Nano.

[156] Júnior R.F.D.A., Lira G.A., Schomann T., Cavalcante R.S., Vilar N.F., de Paula R.C.M., Gomes R.F., Chung C.K., Jorquera-Cordero C., Vepris O. (2023). Retinoic Acid-Loaded PLGA Nanocarriers Targeting Cell Cholesterol Potentialize the Antitumour Effect of PD-L1 Antibody by Preventing Epithelial-Mesenchymal Transition Mediated by M2-TAM in Colorectal Cancer. Transl. Oncol..

[157] Won J.E., Byeon Y., Wi T.I., Lee C.M., Lee J.H., Kang T.H., Lee J.W., Lee Y., Park Y.M., Han H.D. (2022). Immune Checkpoint Silencing Using RNAi-Incorporated Nanoparticles Enhances Antitumor Immunity and Therapeutic Efficacy Compared with Antibody-Based Approaches. J. Immunother. Cancer.

[158] Lee C.K., Atibalentja D.F., Yao L.E., Park J., Kuruvilla S., Felsher D.W. (2022). Anti-PD-L1 F(Ab) Conjugated PEG-PLGA Nanoparticle Enhances Immune Checkpoint Therapy. Nanotheranostics.

[159] Yang W., Pan X., Zhang P., Yang X., Guan H., Dou H., Lu Q. (2023). Defeating Melanoma Through a Nano-Enabled Revision of Hypoxic and Immunosuppressive Tumor Microenvironment. Int. J. Nanomed..

[160] Lu X., Miao L., Gao W., Chen Z., McHugh K.J., Sun Y., Tochka Z., Tomasic S., Sadtler K., Hyacinthe A. (2020). Engineered PLGA Microparticles for Long-Term, Pulsatile Release of STING Agonist for Cancer Immunotherapy. Sci. Transl. Med..

[161] Shubhra Q.T.H., Guo K., Liu Y., Razzak M., Serajum Manir M., Moshiul Alam A.K.M. (2021). Dual Targeting Smart Drug Delivery System for Multimodal Synergistic Combination Cancer Therapy with Reduced Cardiotoxicity. Acta Biomater..

[162] He H., Markoutsa E., Zhan Y., Zhang J., Xu P. (2017). Mussel-Inspired PLGA/Polydopamine Core-Shell Nanoparticle for Light Induced Cancer Thermochemotherapy. Acta Biomater..

[163] Brandhonneur N., Boucaud Y., Verger A., Dumait N., Molard Y., Cordier S., Dollo G. (2021). Molybdenum Cluster Loaded PLGA Nanoparticles as Efficient Tools against Epithelial Ovarian Cancer. Int. J. Pharm..

[164] Guan X., Xu X., Tao Y., Deng X., He L., Lin Z., Chang J., Huang J., Zhou D., Yu X. (2024). Dual Targeting and Bioresponsive Nano-PROTAC Induced Precise and Effective Lung Cancer Therapy. J. Nanobiotechnol..

[165] Wang Y., Hu C., Li Y., Chen Z.-S., Zhang L. (2025). Advancing Cancer Treatment: Innovative Materials in PDT and Diagnostic Integration. Int. J. Nanomed..

[166] Alvarez N., Sevilla A. (2024). Current Advances in Photodynamic Therapy (PDT) and the Future Potential of PDT-Combinatorial Cancer Therapies. Int. J. Mol. Sci..

[167] Huis In ’t Veld R.V., Ritsma L., Kleinovink J.W., Que I., Ossendorp F., Cruz L.J. (2020). Photodynamic Cancer Therapy Enhances Accumulation of Nanoparticles in Tumor-Associated Myeloid Cells. J. Control. Release.

[168] Dai J., Wu M., Xu Y., Yao H., Lou X., Hong Y., Zhou J., Xia F., Wang S. (2023). Platelet Membrane Camouflaged AIEgen-Mediated Photodynamic Therapy Improves the Effectiveness of Anti-PD-L1 Immunotherapy in Large-Burden Tumors. Bioeng. Transl. Med..

[169] Agostinis P., Berg K., Cengel K.A., Foster T.H., Girotti A.W., Gollnick S.O., Hahn S.M., Hamblin M.R., Juzeniene A., Kessel D. (2011). Photodynamic Therapy of Cancer: An Update. CA Cancer J. Clin..

[170] Rezaei S., Fonteyne M., Cruz L., Badr N., Manelkar A., Saberi M., Van Vlierberghe R., Vahrmeijer A., Eich C., Albericio F. (2025). Targeting Rectal Cancer Using Fluorescent Nanoparticles Conjugated to a Novel GRPR-Binding Peptide for Tumor Imaging Applications. Mater. Des..

[171] Chen Q., Xu L., Liang C., Wang C., Peng R., Liu Z. (2016). Photothermal Therapy with Immune-Adjuvant Nanoparticles Together with Checkpoint Blockade for Effective Cancer Immunotherapy. Nat. Commun..

[172] Huis in ‘t Veld R.V., Da Silva C.G., Jager M.J., Cruz L.J., Ossendorp F. (2021). Combining Photodynamic Therapy with Immunostimulatory Nanoparticles Elicits Effective Anti-Tumor Immune Responses in Preclinical Murine Models. Pharmaceutics.

[173] Yang X., Zhang W., Jiang W., Kumar A., Zhou S., Cao Z., Zhan S., Yang W., Liu R., Teng Y. (2021). Nanoconjugates to Enhance PDT-Mediated Cancer Immunotherapy by Targeting the Indoleamine-2,3-Dioxygenase Pathway. J. Nanobiotechnol..

[174] Liao T., Zeng Y., Xu W., Shi X., Shen C., Du Y., Zhang M., Zhang Y., Li L., Ding P. (2025). A Spatially Resolved Transcriptome Landscape during Thyroid Cancer Progression. Cell Rep. Med..

[175] Zhang H., Xu J., Gao B., Wang H., Huang J., Zhou J., Yang R., Yan F., Peng Y. (2021). Synergistic Cascade Strategy Based on Modifying Tumor Microenvironment for Enhanced Breast Cancer Therapy. Front. Pharmacol..

[176] Yang S., Gao H. (2017). Nanoparticles for Modulating Tumor Microenvironment to Improve Drug Delivery and Tumor Therapy. Pharmacol. Res..

[177] Jia H., Chen X., Zhang L., Chen M. (2025). Cancer Associated Fibroblasts in Cancer Development and Therapy. J. Hematol. Oncol..

[178] Yu B., Shao S., Ma W. (2025). Frontiers in Pancreatic Cancer on Biomarkers, Microenvironment, and Immunotherapy. Cancer Lett..

[179] Nia H.T., Munn L.L., Jain R.K. (2020). Physical Traits of Cancer. Science.

[180] Linderman S.W., DeRidder L., Sanjurjo L., Foote M.B., Alonso M.J., Kirtane A.R., Langer R., Traverso G. (2025). Enhancing Immunotherapy with Tumour-Responsive Nanomaterials. Nat. Rev. Clin. Oncol..

[181] Deepak K.G.K., Vempati R., Nagaraju G.P., Dasari V.R., Rao D.N., Malla R.R. (2020). Tumor Microenvironment: Challenges and Opportunities in Targeting Metastasis of Triple Negative Breast Cancer. Pharmacol. Res..

[182] Nabavizadeh A., Narsinh K., Kaufmann T.J., Liu H., Pouliopoulos A.N., Prada F., Agarwal V., Ellingson B.M., Sanvito F., Everson R.G. (2025). Focused Ultrasound in Brain Tumors: Mechanisms, Imaging Guidance, and Emerging Clinical Applications. Am. J. Neuroradiol..

[183] Quan L., Wang M., Wang Z., Du Z. (2025). LIFU (Low-Intensity Focused Ultrasound) Activated Tumor-Starvation/Oxidative-Stress Combined Therapy for Treating Retinoblastoma. Int. J. Nanomed..

[184] Wu N., Zhang Q., Tang R., Deng L., Cao Y., Fu B., Dong H., Huang Z., Wan L., He H. (2025). Ultrasound Visualization of Spatiotemporal Autophagy-Regulated Nanodroplets for Amplifying ICB in Melanoma via Remodeling Tumor Inflammatory Microenvironment. ACS Appl. Mater. Interfaces.

[185] CEBIOTEX (2026). First-in-Man Clinical Trial to Assess Safety and Tolerability of CEB-01 PLGA Membrane in Patients With Recurrent or Locally Advanced Retroperitoneal Soft Tissue Sarcoma After Surgery. https://clinicaltrials.gov/study/NCT04619056.

[186] Price K.A.R., Harrington K.J., Worden F.P., Mesia R., Even C., Schaaf D., Jones S., Saba N.F., Haddad R.I. (2025). VERSATILE-003: A Phase 3, Randomized, Open-Label Trial of PDS0101 and Pembrolizumab Compared with Pembrolizumab for First-Line Treatment of Patients with HPV16-Positive Recurrent/Metastatic Head and Neck Squamous Cell Carcinoma. J. Clin. Oncol..

[187] Jani K., Kaushal N. (2023). Clinical Translation of PLGA Nanoparticles into Market—From Benchside to Breakthrough Therapy. Poly (Lactic-Co-Glycolic Acid)(PLGA) Nanoparticles for Drug Delivery.

[188] Zaccariotto G.D.C., Bistaffa M.J., Zapata A.M.M., Rodero C.F., Coelho F., Quitiba J.V.B., Lima L.L.P.D., Sterman R.G., Cardoso V.M.D.O., Zucolotto V. (2025). Cancer nanovaccines: Mechanisms, design principles, and clinical translation. ACS Nano.

[189] Creemers J.H.A., Pawlitzky I., Grosios K., Gileadi U., Middleton M.R., Gerritsen W.R., Mehra N., Rivoltini L., Walters I., Figdor C.G. (2021). Assessing the Safety, Tolerability and Efficacy of PLGA-Based Immunomodulatory Nanoparticles in Patients with Advanced NY-ESO-1-Positive Cancers: A First-in-Human Phase I Open-Label Dose-Escalation Study Protocol. BMJ Open.

[190] Watson M.J., Vignali P.D.A., Mullett S.J., Overacre-Delgoffe A.E., Peralta R.M., Grebinoski S., Menk A.V., Rittenhouse N.L., DePeaux K., Whetstone R.D. (2021). Metabolic Support of Tumour-Infiltrating Regulatory T Cells by Lactic Acid. Nature.

[191] Kumagai S., Koyama S., Itahashi K., Tanegashima T., Lin Y.-T., Togashi Y., Kamada T., Irie T., Okumura G., Kono H. (2022). Lactic Acid Promotes PD-1 Expression in Regulatory T Cells in Highly Glycolytic Tumor Microenvironments. Cancer Cell.

[192] Zong Z., Xie F., Wang S., Wu X., Zhang Z., Yang B., Zhou F. (2024). Alanyl-tRNA Synthetase, AARS1, Is a Lactate Sensor and Lactyltransferase That Lactylates P53 and Contributes to Tumorigenesis. Cell.

[193] Feng Q., Liu Z., Yu X., Huang T., Chen J., Wang J., Wilhelm J., Li S., Song J., Li W. (2022). Lactate Increases Stemness of CD8 + T Cells to Augment Anti-Tumor Immunity. Nat. Commun..

[194] Wang Z., Dai Z., Zhang H., Liang X., Zhang X., Wen Z., Luo P., Zhang J., Liu Z., Zhang M. (2023). Tumor-Secreted Lactate Contributes to an Immunosuppressive Microenvironment and Affects CD8 T-Cell Infiltration in Glioblastoma. Front. Immunol..

[195] Cheng H., Qiu Y., Xu Y., Chen L., Ma K., Tao M., Frankiw L., Yin H., Xie E., Pan X. (2023). Extracellular Acidosis Restricts One-Carbon Metabolism and Preserves T Cell Stemness. Nat. Metab..

[196] Wang K., Zhang Y., Chen Z.-N. (2023). Metabolic Interaction: Tumor-Derived Lactate Inhibiting CD8^+^ T Cell Cytotoxicity in a Novel Route. Signal Transduct. Target. Ther..

